# Exploring lncRNAs associated with human pancreatic islet cell death induced by transfer of adoptive lymphocytes in a humanized mouse model

**DOI:** 10.3389/fendo.2023.1244688

**Published:** 2023-11-01

**Authors:** Md Munir Hossain, Regan Roat, Jenica Christopherson, Colette Free, Ansarullah, Brian James, Zhiguang Guo

**Affiliations:** ^1^ The Sanford Project/Children Health Research Center, Sanford Research, Sioux Falls, SD, United States; ^2^ Department of Animal Breeding and Genetics, Bangladesh Agricultural University, Mymensingh, Bangladesh; ^3^ Discovery Genomics, Inc., Irvine, CA, United States

**Keywords:** lncRNA, ß-cell, pancreatic islet, T1D, T2D, humanized mouse

## Abstract

**Background:**

Long noncoding RNA (lncRNA)-mediated posttranscriptional and epigenetic landscapes of gene regulation are associated with numerous human diseases. However, the regulatory mechanisms governing human β-cell function and survival remain unknown. Owing to technical and ethical constraints, studying the direct role of lncRNAs in β-cell function and survival in humans *in vivo* is difficult. Therefore, we utilized humanized mice with human islets to investigate lncRNA expression using whole transcriptome shotgun sequencing. Our study aimed to characterize lncRNAs that may be crucial for human islet cell function and survival.

**Methods:**

Human β-cell death was induced in humanized mice engrafted with functional human islets. Using these humanized mice harboring human islets with induced β-cell death, we investigated lncRNA expression through whole transcriptome shotgun sequencing. Additionally, we systematically identified, characterized, and explored the regulatory functions of lncRNAs that are potentially important for human pancreatic islet cell function and survival.

**Results:**

Human islet cell death was induced in humanized mice engrafted with functional human islets. RNA sequencing analysis of isolated human islets, islet grafts from humanized mice with and without induced cell death, revealed aberrant expression of a distinct set of lncRNAs that are associated with the deregulated mRNAs important for cellular processes and molecular pathways related to β-cell function and survival. A total of 10 lncRNA isoforms (SCYL1-1:22, POLG2-1:1, CTRB1-1:1, SRPK1-1:1, GTF3C5-1:1, PPY-1:1, CTRB1-1:5, CPA5-1:1, BCAR1-2:1, and CTRB1-1:4) were identified as highly enriched and specific to human islets. These lncRNAs were deregulated in human islets from donors with different BMIs and with type 2 diabetes (T2D), as well as in cultured human islets with glucose stimulation and induced cell death induced by cytokines. Aberrant expression of these lncRNAs was detected in the exosomes from the medium used to culture islets with cytokines.

**Conclusion:**

Islet-enriched and specific human lncRNAs are deregulated in human islet grafts and cultured human islets with induced cell death. These lncRNAs may be crucial for human β-cell function and survival and could have an impact on identifying biomarkers for β-cell loss and discovering novel therapeutic targets to enhance β-cell function and survival.

## Introduction

Type 1 diabetes (T1D) is an autoimmune disorder characterized by the destruction of insulin-producing β cells in the pancreas. Its progression involves complex interactions between genetic, environmental, and immunological factors (1). Genetic factors play a significant role in the progression of T1D, influencing both susceptibility and disease severity. A study conducted by Redondo et al. in 2021 explored the genetic regulation of T1D progression, shedding light on the key genes and pathways involved in disease development (2). To date, many studies identified numerous genetic variants, along with immunological processes and environmental triggers, associated with T1D progression, providing valuable insights into the underlying mechanisms that drive the course of this autoimmune condition (1, 2). Understanding the genetic basis of T1D progression is crucial for developing more targeted and personalized approaches to its management and treatment. Pancreatic β cells have a pivotal role in regulating glucose homeostasis by releasing insulin in response to elevated blood glucose level, subsequently initiating the uptake of glucose by the target tissues. The lack of β cells due to autoimmune destruction through the infiltration of dendritic cells, macrophages, and T lymphocytes results in absolute insulin deficiency and leads to T1D (3). In another form, the inability of β cells to synthesize or increase insulin levels sufficiently to stimulate glucose uptake in the face of insulin resistance leads to type 2 diabetes (T2D) (4). In both cases, the loss and/or inability of β cells lead to the dysfunctional regulation of blood glucose, an abrupt onset of symptoms, and dependency on exogenous insulin to sustain life. Recently, considerable efforts have been made to elucidate the non-coding RNA (ncRNA)-mediated molecular mechanisms underlying β-cell failure. The integration of transcriptional and chromatin maps, along with in-depth analysis of RNA-Seq from human pancreatic β cells, identified thousands of intergenic and antisense islet-cell long non-coding RNAs (lncRNAs) that are dynamically regulated as an integral component of β-cell differentiation as well as maturation. Some of them were identified as deregulated in T2D (5).

LncRNAs are like mRNA transcripts that are grouped and characterized based on the fact that they are 200 nt to more than 100 kilobases (kb) in length, with little or no protein coding potential (6), often spliced, may be polyadenylated, transcribed by RNA polymerases from either strand within a coding locus, and may be located either within the nucleus or cytoplasm (7, 8). LncRNAs are not as highly conserved as miRNAs and other medium ncRNAs at the primary sequence level, and it is estimated that there are ~17,000 in the human and ~10,000 in the mouse genome (9). Most lncRNAs exhibit a low degree of evolutionary conservation compared to protein coding genes and therefore evolve very differently. Some lncRNAs are found to be widely distributed in different tissues while most of them are expressed in a more tissue- or cell-type specific fashion than mRNA genes (10, 11). Through the analysis of approximately 8,000 lncRNAs in 24 different tissues and cell types, it has been shown that the tissue specificity of the number of lncRNAs is about four times (78%) greater than that of the number of protein coding genes (19%) (10).

Although understanding the function of lncRNAs is only beginning to emerge, there is already some strong evidence of their involvement in different cellular processes leading to the development of several diseases. LncRNAs play a variety of essential roles in cellular development and metabolism, including regulation of the cell cycle, transcription, splicing, mRNA decay, translation, genetic imprinting, genome rearrangement, and chromatin modification (12–15). They are also essential in association with the chromatin remodeling complex for the specification or silencing of target genes (16–19). In addition, they serve as scaffolds to support multiple proteins to form ribonucleoprotein (RNP) complexes, which stabilize nuclear structures or signaling complexes acting on chromatin and determine histone modifications (11, 15, 20).

Recent genome-wide association studies revealed a substantial number of candidate lncRNAs important in different diseases and physiology. For example, lncRNA ANRIL, located in the p15/CDKN2B-p16/CDKN2A-p14/ARF, has been shown through genome-wide association studies to be genetically associated with diverse diseases such as diabetes, cancer, and heart disease (21, 22). The significance of lncRNAs in various aspects of β-cell biology, including proliferation, failure, insulin secretion, and apoptosis, has been extensively emphasized through numerous studies, primarily conducted in mice. In a model of diet-induced obesity-associated T2D, deregulation of lncRNAs has been observed, and certain alterations have been proposed to contribute to the failure of β cells during disease progression (23). The downregulation of lncRNA HOTTIP in db/db and C57BL/6J mice has been suggested to hinder insulin secretion and disrupt the cell cycle in β cells through the MEK/ERK pathway (24). Another study conducted in db/db mice and MIN6 cells has revealed that lncRNA Malat1 potentially influences β-cell dysfunction in diabetic conditions by modulating the Malat1/Ptbp1/PKM2 pathway (25). Interestingly, lncRNA ANRIL has been shown to be differentially expressed in dysfunctional β cells *in vitro* (26). Depletion of β-cell-specific lnRNA HI-LNC25 has been shown to downregulate GLIS3 mRNAs (related to diabetes) and thus demonstrates a gene regulatory function of lncRNAs in pancreatic islets. In addition, dysregulation of lncRNAs has also been observed in T2D and subsequently mapped to genetic loci underlying diabetes susceptibility (5). Using ChIP and RNA sequencing analysis, 12 β-cell–specific and 5 α-cell-specific lncRNAs were revealed (27). LncRNA PINK1, antisense to the PTEN Induced Putative Kinase 1 gene (PINK1-AS), could be induced by the important inhibitor of insulin through signaling Phosphatase and Tensin homolog (PTEN). Depletion of lncRNA-PINK1 is associated with T2D (28). Singer et al. (2019) delve into the dysregulated glucagon secretion and its implications for diabetes, showcasing how specific lncRNAs, such as Paupar, play a vital role in α-cell development and function, providing a distinct mechanism for glucose homeostasis (29). β-cell-specific lncRNAs and their dynamic regulation during differentiation and changing glucose concentrations have been studied (30). It unravels their involvement in β-cell gene regulation and hints at their role in diabetes, providing insights into lncRNA function through transcript knockdowns and co-expression network analysis. A study has also showcased how lncRNAs like HASTER maintain cell-specific physiological concentrations, revealing a unique regulatory element affecting transcription and causing diabetes mellitus when disrupted (31).

Because of the existence of lncRNAs and their regulation in pancreatic islets as shown in different studies, it is likely that many lncRNAs may affect different aspects of islet physiology, maintenance of β-cell functions, and survival. However, as there is limited conservation across different species, many of the candidate lncRNAs identified as important in β-cell physiology in mice do not have human orthologs. Relying solely on computational tools that utilize sequence alignments may not always be suitable for accurately identifying mouse lncRNA orthologs in humans. Therefore, conducting studies using human pancreatic islets/β cells *in vivo* is crucial to identify the significance of lncRNAs in human β-cell biology. Owing to technical and ethical constraints, it is challenging to directly study the role of lncRNAs in β-cell function and survival in humans. Thus, a humanized mouse model with functional human islets, mimicking the setting of *in situ* functional human islets, is a valuable tool for studying human lncRNAs through a systematic identification, characterization, expression profiling, and exploration of regulatory functions of lncRNAs during loss of human β-cell function and survival. We previously used humanized mice with transplanted human islets to study β-cell function and regeneration (32–37). Adoptive lymphocyte transfer (ALT) has found applications not only in the study of tolerance induction mechanisms in transplantation but also in understanding autoimmune processes that lead to the destruction of beta cells in mouse models. Thus, to generate the humanized mice, human pancreatic islets isolated from deceased donors were transplanted under the kidney capsule of streptozotocin (STZ)-induced diabetic immunodeficient NOD.scid mice to achieve normoglycemia. Since adoptive transfer of lymphocytes from diabetic NOD mice can induce β-cell death in NOD.scid mice (33, 38–40), we carried out adoptive lymphocyte transfer (ALT) to induce human β-cell death in humanized mice engrafted with functional human islets, for studying the existence, regulation, and potential role of lncRNAs in human islet cell function and survival. We explored the repertoires of lncRNAs specific to human islets, their deregulation during the loss of human pancreatic islet cell death, and provided the possibility to identify them as potential biomarkers for β-cell death. In addition, the selected lncRNAs may represent important future therapeutic targets for treating diabetes.

## Materials and methods

### Experimental animals

All experiments were performed in accordance with the protocol reviewed and approved by the Institutional Animal Care and Use Committee at Sanford Research (protocol 77-08-16D). NOD.scid and NOD.shiLt mice at 8 weeks of age were purchased from the Jackson Laboratory (Bar Harbor, ME, USA) and housed in a pathogen-free animal facility on a 12-h light:dark cycle with free access to food and water. NOD.scid mice were injected with streptozotocin (STZ) (Sigma Aldrich, St Louis, USA) intraperitoneally at 180 mg/kg to induce diabetes. Diabetes was diagnosed when non-fasting blood glucose level was >400 mg/dL on two consecutive measurements using the GLUCOMETER ELITE blood glucose monitoring system (Bayer Co, Elkhart, IN, USA). Blood glucose and body weight were then monitored twice weekly, and all mice received daily insulin treatment prior to human islet transplantation. Diabetic NOD mice were used as the source of lymphocytes for the adoptive lymphocyte transfer. Unless otherwise specified, all types of samples were analyzed from three biological replicates in triplicate conditions as technical replicates (e.g., islets from three human donors, each tested in triplicate).

### Human islets and transplantation

Human islets were obtained through the Integrated Islet Distribution Program (IIDP, http://iidp.coh.org) sponsored by the National Institute of Diabetes and Kidney and Digestive Diseases (NIDDK). Since islets were isolated from cadaveric donors and no living individuals were involved, our study did not meet the definition of research with human subjects. Sanford Health Institutional Review Board had reviewed our study and documented that our study did not meet the regulatory requirements for human subject research. Islet purity and morphology were assessed using Dithizone (DTZ) staining. Islets from three non-T2D donors (BMI 25.1 ± 2.0) with purity >85% and viability >95% were used for transplantation. Characteristics of the donor and properties of islets used for transplantation as well as for other experimental purposes are listed in **Table S10** in **Supplementary Materials**. Streptozotocin-induced diabetic NOD.scid mice were transplanted with 3,000 islet equivalents (IEQs) under the left kidney capsule as previously described (32, 33). Following transplantation, blood glucose and body weight were monitored daily. Reversal of diabetes to normoglycemia was defined as blood glucose <200 mg/dL at which point blood glucose and body weight were monitored twice weekly.

### Adoptive lymphocyte transfer to induce β-cell apoptosis

Lymphocytes (1 × 10^6^) from the diabetic female NOD mice with blood glucose >400 mg/dL were adoptively transferred via tail vein injection to transplanted mice bearing human islets with normoglycemia. For each of the three sets of donor islets, at least three mice received lymphocytes (ALT) and at least three received PBS as a control. Blood glucose and body weight were monitored daily, and blood and human islet grafts were collected immediately once the blood glucose of ALT-treated mice reached >200 mg/dL for two consecutive blood glucose measurements taken 30 min apart. Blood and human islet grafts of the control mice were collected concurrently.

### Collection and processing of tissue and cells

Blood from the humanized NOD.scid mice was collected via cardiac puncture and plasma was separated. Peripheral blood mononuclear cells (PBMCs) were separated from the blood collected from streptozotocin-induced diabetic NOD.shiLt mice using histopack-1077 (Sigma, MO, USA) according to the standard protocols and resuspended in 700 μl of QIAzol lysis reagent (QIAGEN GmbH, Hilden, Germany) for isolation of total RNA. Spleen from the diabetic NOD.shiLt mice was collected in RPMI medium 1640 (with L-glutamine) (Life Technologies, CA, USA), homogenized, and filtered. The cell suspension was centrifuged to pellet the lymphocytes and then resuspended in 700 μl of QIAzol for isolation of total RNA. The left kidney bearing the human islet graft was removed from the mouse and the graft was sliced into two halves using a scalpel blade. One-half of the graft left remained attached to the mouse kidney and was fixed in 4% PFA for histological studies. The other half of the human islet graft was peeled from the kidney, excess kidney capsule was removed, and it was washed twice in PBS to remove any single-cell contamination. Kidney from the humanized NOD.scid mice and half of the islet grafts from ALT-treated or PBS-treated mice were placed in a 2.0-mL microcentrifuge tube prefilled with zirconium bead (1.0 mm) (Benchmark, D1032-15) and 700 μl of QIAzol. Tissues and cells in QIAzol were homogenized in a high-throughput tissue homogenizer Precellys®24 (Bertin Technologies, MD, USA) and total RNA was isolated.

### Isolation of RNA and quality control

Total RNA from ALT-treated and PBS-treated human islet grafts, isolated/cultured human islets (1,000 IEQ), and mouse tissues/cells resuspended and homogenized in QIAzol (QIAGEN) was isolated using the miRNeasy Micro Kit (QIAGEN GmbH, Hilden, Germany) according to the manufacturer’s recommendations. Separate fractions of nuclear and cytoplasmic total RNA from islets were isolated using the Cytoplasmic and Nuclear RNA Purification Kit (Norgen Biotek Corporation, Thorold, Canada) according to manufacturer’s instructions. Genomic DNA contamination was removed from the RNA samples using the TURBO DNA-free™ kit (Ambion, Foster City, CA, USA). Total RNA from adult human tissues was obtained from Agilent Technologies (**Table S10** in **Supplementary Materials**). The concentration of the RNA was analyzed using the Nanodrop 8000 Spectrophotometer (Thermo Fisher Scientific Inc, DE, USA). The RNA integrity and quality of RNA were evaluated using the Agilent 2100 Bioanalyzer with RNA 6000 Nano reagents and Chips Kit (Agilent Technologies Inc, CA, USA).

### Whole transcriptome sequencing and analysis of sequences

Total RNA isolated from pooled ALT-treated human islet grafts, PBS-treated human islet grafts, and the corresponding isolated human islets that were used for transplantation were utilized for whole transcriptome sequencing. The total RNA samples were ribo-depleted using the Ribominus Eukaryote System (Life Technologies), and barcoded fragment sequencing libraries were constructed using the Ion Total Rna-Seq Kit V2 (Life Technologies). Three libraries per Ion Proton PI Chip were sequenced on the Ion Proton sequencer using 200-bp sequencing reagents. Reads aligning with Bowtie2 (http://tophat.cbcb.umd.edu/) to adaptor, repetitive sequences, ribosomal sequences, and other small transcripts were removed. The remaining reads were aligned to the human genome (hg19) with a modified two-step alignment method according to https://www.sevenbridges.com/ion-proton-rna-seq-alignment/#f6 and http://ioncommunity.lifetechnologies.com/docs/DOC-7062 that uses a combination of Tophat2, and Bowtie2 (http://tophat.cbcb.umd.edu/). Differential expression analysis of transcript was determined using the Cufflinks package with the Cuffdiff program (http://cufflinks.cbcb.umd.edu/) according to the manual (http://cufflinks.cbcb.umd.edu/manual.html#cuffdiff). For normalization of differential expression, the geometric option was used and FPKM values were scaled via the median of the geometric means of fragment counts across all libraries, as described in (41). In order to be considered differentially expressed, a *q*-value (false discovery rate-adjusted *p*-value of the test statistic) ≤0.05, a fold change ≥2, and a Cuffdiff “Test status” of OK were required. Transcripts were annotated with a combination of Ensembl release 75 and LNCipedia version 3.0 (www.lncipedia.org/).

### Reverse transcription and SYBR green qPCR for lncRNAs and mRNAs

Reverse transcription of 200 ng of total RNA from each sample was performed using Superscript III First strand Synthesis SuperMix for qRT-PCR (Invitrogen, Carlsbad, CA, USA) containing oligo (dT)_20_, and random hexamers. The cDNA was stored at −20°C until use. Primers for lncRNAs and mRNAs were designed using the Primer Express 2.0 software program (Applied Biosystems, Foster City, CA, USA) and PrimerQuest tools (https://www.idtdna.com/Primerquest/Home/Index). Primers for lncRNAs were designed from the exon portion, ensuring no matching to the mRNA sequence. Selected primer pairs for each lncRNAs were checked for their specificity by BLAST search for short nucleotide sequence against the *Homo sapiens* (Hg19) reference transcriptome and genome assembly as well as the Primer-BLAST tool (http://www.ncbi.nlm.nih.gov/tools/primer-blast/index.cgi?LINK_LOC = BlastHome). A list of the primers and sequences is presented in **Table S11** in **Supplementary Materials**. Primers were synthesized by Integrated DNA Technologies. All the primers utilized were optimized in order to ensure optimal reaction efficiencies for both target and housekeeping reference genes (GAPDH and ACTB). The specificity and identity of each primer pair were further confirmed by sequencing (Eurofins MWG operon, AL, USA). Immediately before use, cDNA was diluted and 1 µL of the diluted cDNA was used per 20 µL of qRT-PCR reaction. Triplicate reactions were performed for each gene by the standard PCR protocol with a 20-µL reaction volume consisting of 10 µL of Platinum SYBR Green qPCR SuperMix-UDG with ROX (Invitrogen), forward and reverse primers at 0.2 µM final concentration, and 1 µL of diluted template cDNA. A universal thermal cycling parameter specified for the instrument (ABI 7500 real-time PCR system, Applied Biosystems, CA, USA) was 50°C for 2 min, 95°C for 2 min, followed by 40 amplification cycles at 95°C for 15 s and 60°C for 30 s. In addition, at the end of the last cycle, a dissociation curve was generated by starting the fluorescence acquisition at 60˚C and taking measurements at every 7-s interval until the temperature reached 95˚C. The same PCR protocol was used for all primers. The data were normalized against the geometric mean of GAPDH and ACTB in each case (except for the fact that GAPDH only was used as endogenous control for exosomal RNA), and subsequent analysis was performed using ΔΔCt methods (42). The *p*-values were calculated based on a Student’s *t*-test of the replicate 2^−ΔΔCt^ values for each gene in the control group and treatment groups. A probability of *p* ≤ 0.05 was considered to be significantly differentially expressed.

### Combined TUNEL and immunofluorescent labeling

Terminal deoxynucleotidyl transferase (TdT)-mediated dUTP nick-end labeling (TUNEL) assay coupled with immunofluorescent labeling of insulin was performed on formalin-fixed paraffin-embedded (FFPE) human islet graft sections. Briefly, sections were deparaffinized in Citrisolv (Fisher Scientific, PA, USA), gradually rehydrated in a series of EtOH, post fixed in 4% PFA, and treated with 0.5 N HCl. Following 30 min blocking in Donkey serum, DNA labeling was performed at 37°C for 1 h using the APO BrdU TUNEL assay kit (Life Technologies) according to the manufacturer’s instructions. Sections were incubated with Alexa Fluor 488-conjugated mouse anti-BrdU and Guinea pig anti-insulin (Abcam, MA, USA) overnight at 4°C. Cy3 conjugated Donkey Anti-Guinea pig IgG was used to detect insulin-positive cells. DAPI was used for nuclear counterstaining. Apoptosis was analyzed by counting TUNEL-positive β cells in each islet graft.

### *In vitro* culture of human islets and exposure to glucose of different concentrations and cytokines

Human islets were cultured *in vitro* in triplicate for 60 h in glucose-free RPMI 1640 media supplemented with either 4 mM or 11 mM glucose to identify the lncRNAs’ regulation in response to glucose-mediated stimulation according to the method described earlier (5). For the cytokine treatment, human islets from three donors were cultured free-floating in groups of 300 islets per 60 mm Corning^®^ Ultra-low attachment culture dishes (Sigma-Aldrich) in CMRL1066- without L-glutamine and phenol red (Corning Celgro, VA, USA), supplemented with 10% exosome-depleted FCS, benzylpenicillin (100 U/mL), and streptomycin (0.1 mg/mL). After 12 h of culture, the medium was supplemented with a mixture of cytokines (IL-1β, 50 IU/mL; IFN-γ, 1,000 IU/mL; and TNF-α, 1,000 IU/mL). Control islets were incubated under identical conditions without (w/o) inflammatory mediators. Each treatment from each donor was performed in triplicate. Islets with the cytokine mix were cultured for 48 h. At the end of culture, both cytokine-treated and control islets were washed twice in cold PBS and then one-third of the harvested islets were lysed in QIAzol for isolation of RNA, one-third were fixed in 4% PFA for immunofluorescence coupled with TUNEL assay, and the remaining one-third were used for fluorescence-activated cell sorting for quantification of apoptotic cells. Islets in QIAzol were used to isolate total RNA, as described earlier, using the miRNeasy Micro Kit (QIAGEN GmbH, Hilden, Germany) according to the manufacturer’s instructions.

### Preparation and staining of exosomes

Culture media was collected and centrifuged at 3,000 × *g* for 15 min to remove cells and cell debris. The collected supernatant was mixed with ExoQuick-TC Exosome Precipitation Solution (System Biosciences, Mountain View, CA) in a 5:1 ratio and incubated 12 h at 4˚C. After incubation, the media was centrifuged twice at 1,500 × *g* for 30 and 5 min, respectively, to remove the supernatant. The pellet containing exosomes was resuspended in 100 μL of 1× Annexin V Binding Buffer from the FITC Annexin V Apoptosis Detection kit (BD Biosciences, CA, USA) and stained for microscopy. For immunostaining the exosomes, 5 μL of Annexin V-FITC solution, 5 μL of Vybrant DiI from Vybrant Cell-Labeling Solutions (Molecular Probes, OR, USA), and 1 μL of DAPI dilactate (Molecular Probes, OR, USA) were added to the resuspended pellet, mixed, and incubated at 37°C for 15 min. Immediately after incubation, the solution was centrifuged at 3000 × *g* for 15 min; the supernatant was removed and washed twice with PBS. The stained pellet was resuspended in 50 μL of PBS; a small drop was dispersed on microscope slides. Slides were allowed to partially dry, then mounted using Fluoroshield with 1,4 Diazabicylo [2.2.2] octane (Sigma Aldrich (Sigma-Aldrich). Cover glass was fixed using nail polish. Imaging was performed in a Nikon Eclipse Ti confocal microscope equipped with suitable lasers using 100× objectives with immersion oil at 10× zoom.

### TUNEL assay and immunofluorescence of whole mount islets

All incubation and washing of islets were performed in Millicel cell culture inserts, 12.0 μm, 12 mm diameter (Millipore, MA, USA) in a 24-well tissue culture plate. Islets cultured with and without cytokines were washed twice in PBS, then fixed in 4% PFA for 30 min at room temperature. After washing twice in PBS, islets were treated with 0.5 N HCl for 30 min. The remaining procedure was similar to the immunostaining and TUNEL assay that were used for FFPE sections. Islets were mounted using Fluoroshield containing 1,4 Diazabicylo [2.2.2] octane (Sigma-Aldrich) and glass beads (500 μm). The cover glass was fixed to achieve air-tight sealing using nail polish. Z-stack images of whole islets were taken using a 2-μm interval in a Nikon Eclipse Ti confocal microscope, with a 20× objective at 2,048 × 2,048 pixel resolution. The level of apoptosis was calculated by counting the TUNEL (green)-positive β cells (red) in each islet.

### Flow cytometry and detection of apoptosis

Islets cultured with and without cytokine mix were washed twice with PBS. Islet cells were dissociated by sequential incubation with StemPro® Accutase® Cell Dissociation Reagent in order to minimize induction of apoptosis during dissociation. Briefly, islets were incubated with Accutase for 8 min at 37°C, pipetted several times with a Pasteur glass pipette, and allowed to settle for 2 min. Supernatant was transferred to CMRL-1066 supplemented with 10% FCS. This step was repeated with the remaining un-dissociated islets two to three times until most of the islets were dissociated to single cells. Dissociated cells with media were filtered through a cell strainer (35 μm), washed twice with PBS, and resuspended in 1× Annexin V Binding Buffer from the FITC Annexin V Apoptosis Detection kit (BD Biosciences) in a concentration of 1.0 × 10^6^ cells/100 μL. Afterward, the cell suspension (100 μL) was stained with 5 μL of FITC Annexin-V antibody and 5 μL of PI for 15 min at room temperature. Flow cytometry data acquisition was performed in a BD Accuri™ C6 flow cytometer (BD Bioscience) and analyzed with FlowJo software. All samples were analyzed in triplicate from all three donors. Annexin V-positive/PI-negative cells were considered to be early apoptotic cells and Annexin V-positive/PI-positive cells were considered as late apoptotic cells. The rate of cell apoptosis was calculated as the sum of early and late apoptotic cells and compared between islets cultured with and without cytokines.

### Statistics

Unless otherwise specified, all types of samples were analyzed from three biological replicates in triplicate conditions as technical replicates (e.g., islets from three human donors, each tested in triplicate). In order to be considered differentially expressed in RNA-Seq, a *q*-value (false discovery rate-adjusted *p*-value of the test statistic) ≤0.05, fold change ≥2, and a Cuffdiff “Test status” of OK were confirmed. Comparisons of the expression level of mRNAs and lncRNAs in any experiment was performed using Student’s *t*-test of the replicate 2^−ΔΔCt^ values. A probability of *p* ≤ 0.05 was considered to be statistically significantly differentially expressed. Correlation between the expression of mRNAs and closely located lncRNAs was estimated using the Pearson correlation method using GraphPad prism software version 6.0. Significant correlations were considered by calculating two-tailed *p*-values where the value of alpha was 0.05. Significant enrichment of a set of transcripts in a particular molecular network or pathway was predicted using the right-tailed Fisher’s exact test in IPA (Ingenuity Pathway Analysis). Significance level, *p*-values, and other statistical parameters and description were also stated in the respective methodology and results section.

## Results

### Humanized mice as a model of human β-cell death and function

**Figure 1:**
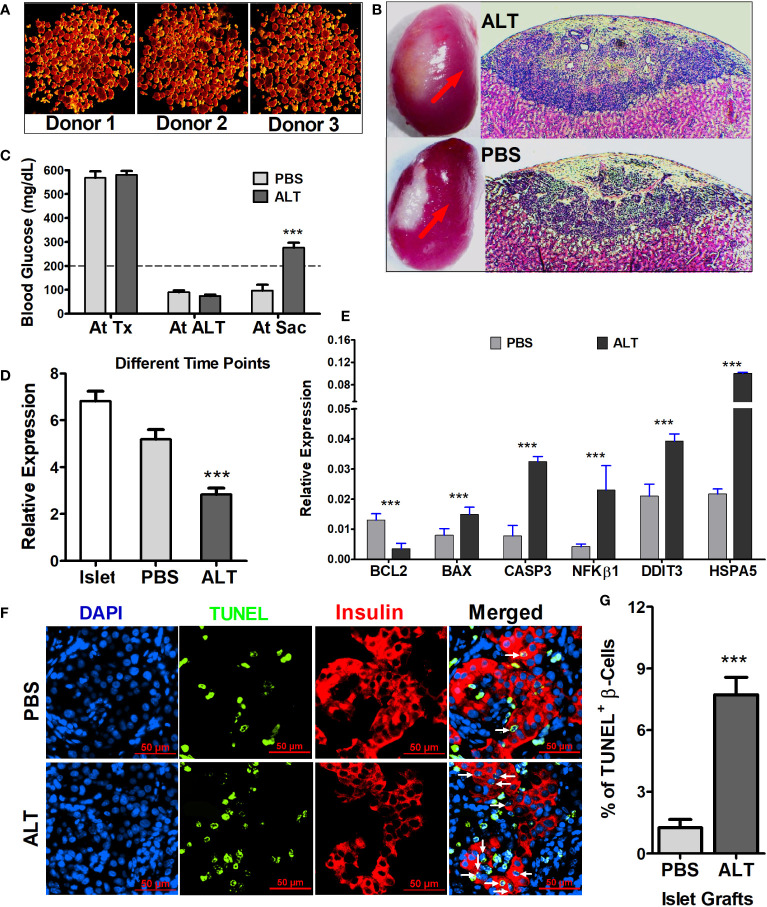
Morphological, cellular, and molecular characterization of isolated islets and islet grafts for the loss of β-cell survival and function. **(A)** Representative images of human islets from three donors stained with dithizone for quality assessment before transplantation and other usages. **(B)** Representative images of mouse kidney engrafted with human islets (red arrow) from ALT-treated and PBS-treated mice at the time of graft collection and H&E-stained sections of islet grafts from ALT-treated (upper panel) and PBS-treated mice (lower panel). **(C)** Average blood glucose level (mg/dL) of all mice used for the study at their different time points (Tx, transplantation; ALT, adoptive lymphocyte transfer; Sac, sacrifice. ***p < 0.01. **(D)** Relative expression of insulin mRNA in the isolated islets from three donors used for transplantation, in PBS- and ALT-treated islet grafts. ***p < 0.01. GAPDH was used as the endogenous control. **(E)** Relative gene expression level of apoptotic markers (BCL2, BAX, NFkB1, and CASP3) and ER stress markers (DDIT3 and HSPA5). (***p < 0.01). GAPDH was used as the endogenous control for the relative quantification. **(F)** Representative images of TUNEL staining (green) coupled with insulin staining for β cells (red) in sections of ALT and PBS-treated islet grafts. Apoptotic β cells were indicted by the white arrows. **(G)** Percentage of apoptotic β cells (TUNEL^+^ β cells) in total β cells in PBS-treated islet grafts and ALT-treated treated islet grafts. (***p < 0.01).

STZ-induced diabetic NOD.scid mice were severely hyperglycemic. Elimination of endogenous pancreatic β cell of mouse origin was confirmed by the blood glucose level before islet transplantation (568.25 ± 26.68 mg/dL and 580.6 ± 16.34 mg/dL in mice used for ALT and PBS control, respectively), as well as by immunohistochemistry at the end of the experiment. Human islets from each donor (*n* = 3), characterized with purity >85% (**Figure 1A**), were transplanted under the left kidney capsule of diabetic mice (**Figure 1B**) to reverse diabetes. Stable normoglycemia (81.1 ± 5.49 mg/dL) for at least 2 weeks ensures success of transplantation and functional regulation of glucose homeostasis in mice by human islets. ALT was then performed to induce islet cell death and loss of function in the humanized mice. Within 8–21 days, ALT-treated mice exhibited a significant increase in blood glucose levels compared to PBS-treated mice (276 ± 20.68 mg/dL versus 96 ± 25.2 mg/dL, *p* < 0.001, **Figure 1C**). Islet grafts collected from both ALT and PBS groups were characterized for β-cell loss/apoptosis, level of insulin, and expression of insulin, apoptotic, and endoplasmic reticulum (ER) stress-related genes. The relative expression of insulin mRNA in ALT-treated islet grafts was significantly lower compared to PBS-treated islet grafts and isolated islets used for transplantation from the same donor (*p* < 0.001, **Figure 1D**). The relative expression of pro-apoptotic transcripts (BAX, CASP3, and NFKβ1) was significantly higher in ALT-treated islet grafts compared to PBS-treated islet grafts. The expression of anti-apoptotic transcript BCL2 was significantly lower in ALT-treated islet grafts. Similarly, the level of ER stress, as determined by the expression levels of DDIT3 and HSPA5, was significantly higher in ALT-treated islet grafts compared to PBS counterparts (**Figure 1E**). The percentage of TUNEL^+^ β cells was significantly higher in ALT-treated islet grafts than in PBS-treated islet grafts (*p* < 0.001, **Figures 1F, G**). Together, these findings suggest that ALT selectively and specifically induces cell death in human pancreatic islets and loss of function in humanized mice.

### Deregulation of transcripts between isolated human islets and islet graft with or without ALT

**Figure 2:**
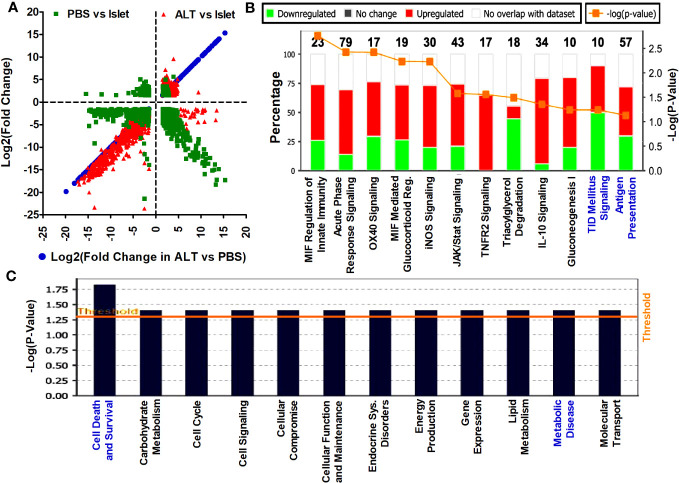
Distribution pattern of differentially expressed mRNA transcripts and the affected signaling pathways. **(A)** The log2(fold change) >1.5 with *p* < 0.05 of mRNAs in ALT vs. PBS were plotted on the *x*-axis and the log2(fold change) in other comparisons are plotted on the *y*-axis to visualize their distribution pattern. **(B)** List and level of enrichment of top canonical pathways identified to be affected by the differentially expressed mRNAs in ALT vs. PBS using the IPA tool. The Canonical Pathways tab displays the most significant Canonical Pathways across the differentially expressed mRNA transcripts in ALT vs. PBS using the IPA tool. The significance value for the canonical pathways was calculated by Fisher’s exact test right tailed. The significance indicates the probability of association of molecules from the deregulated transcripts with the canonical pathway. The base of the log in “-Log(*p*-value)” is 10. **(C)** Disease pathways regulated by the aberrantly expressed mRNAs between ALT-treated islet grafts and PBS-treated islet grafts. Downstream effects from the changes of mRNA transcripts between ALT-treated islet grafts and PBS-treated islet grafts on the biological trends and the affected biological processes and disease were predicted and visualized. The base of the log in “-Log(*p*-value)” is 10.

Following the differential expression analysis, based on the annotation from Ensembl release 75 and LNCipedia version 3.0, expression values of mRNAs and lncRNAs were separated for independent analysis. Annotated Ref seq reads in islets, as well as islet grafts from ALT- and PBS-treated mice, were compared to each other. A total of 62,500 mRNAs transcripts, either with more than a 1.5-fold change or a *p*-value < 0.05 in any group of samples, were included in the analysis. Log2(fold change) of PBS-treated islet grafts vs. islets and ALT-treated islet grafts vs. islets were plotted to show the distribution of patterns and the number of differentially expressed mRNAs in three ways of comparison (**Figure 2A**). Out of 1,729 deregulated mRNAs, 1,182 were significantly downregulated in PBS-treated islet grafts compared to islets. The changes in expression may be due to transplantation and the alteration of the islet environment from the human pancreas to the kidney capsule of mice. More aberrant expression of mRNAs was found in the ALT-treated islet grafts. A total of 1,925 mRNA transcripts were differentially expressed in the ALT-treated islet grafts compared to their islet counterparts. Among these, 933 mRNAs were upregulated and the remaining 992 mRNAs were downregulated. This major deregulation encompasses the cellular changes that occurred in the PBS-treated islet grafts compared to isolated islets, as well as the adoptive lymphocyte-mediated cellular changes. Comparison of expression between ALT-treated islet grafts and their PBS-treated counterparts revealed a differential expression of 844 mRNAs, of which 347 mRNAs were upregulated and 497 mRNAs were downregulated. These are the interesting transcripts that might be associated with the loss of β-cell function and survival due to the immune-mediated destruction of β cells by the adoptively transferred lymphocytes from diabetic mice. The expressions of 12 selected mRNAs were further validated using real-time qRT-PCR (**Figure S1** in **Supplementary Materials**). The list of differentially regulated mRNAs with more than ±1.5-fold change in expression, supported by statistically significant expression data in different comparisons, is presented in the Additional results file.

### Aberrant expression of mRNA transcripts reveals important pathways for human β-cell survival and T1D

Differentially expressed mRNAs in PBS-treated islet grafts and ALT-treated islet grafts compared to islets, as well as ALT-treated islet grafts compared to PBS-treated islet grafts, were analyzed using IPA from Ingenuity® Systems (http://www.ingenuity.com). Differentially expressed mRNAs with a >1.5-fold change and a *p*-value < 0.05 were uploaded to the IPA for core analysis. Several pathways and gene networks represented among the sets of protein-coding mRNAs were identified as differentially regulated in the comparison between islets, PBS-treated islet grafts, and ALT-treated islet grafts. The most enriched molecular networks from aberrantly expressed mRNA between PBS-treated islet grafts and islets were “Death receptor signaling, Endoplasmic reticulum stress pathway, ERK/MAPK, ERK5, IL-8, ILK, iNOS, Jak/Stat, p38 MAPK, Pancreatic adenocarcinoma signaling, and MIF regulation of innate immunity pathways” and comprised 575 differentially expressed transcripts (**Figure S2**; **Table S4** in **Supplementary Materials**). The most affected disease and functional regulation by these aberrantly expressed mRNAs were “Cell death and survival”, and “Cell cycle regulation” (**Figure S3** in **Supplementary Materials**). The 505 differentially expressed mRNAs between ALT-treated islet grafts and islets were found to be associated with the “Assembly of RNA polymerase II complex, Endoplasmic reticulum stress pathways, IL-17A, EIF, ILK signaling, Unfolded protein response, CXR4 signaling pathway, etc.” (**Figure S4**; **Table S5** in **Supplementary Materials**). The only disease and functional regulation by these mRNAs was cell cycle regulation (**Figure S5** in **Supplementary Materials**). Aberrantly expressed mRNAs between ALT-treated islet grafts and PBS-treated islet grafts are our major focus, as the difference might be due to the loss of human β-cell function and survival. IPA of mRNA transcripts deregulated in ALT-treated islet grafts compared to their PBS-treated counterparts revealed several canonical pathways involved in cell death and survival, autoimmunity, and T1D signaling pathways. These are “MIF regulation of innate immunity, Acute phase response, Antigen presentation, T1D signaling, OX40 signaling, iNOS, IFN2R signaling, JAK/Stat, IL-10, Gluconeogenesis I, and Tryacylglycerol degradation pathways” (**Figure 2B**; **Table S1** in **Supplementary Materials**). Interestingly, cellular functions and disease pathways regulated by the aberrantly expressed 357 mRNAs between ALT-treated islet grafts and PBS-treated islet grafts were “Cell death and survival, Carbohydrate metabolism, Cell cycle, Cell signaling, Cellular compromise, Cellular function and maintenance, Endocrine system disorders, Energy production, and Molecular transport” (**Figure 2C**; **Table S2** in **Supplementary Materials**).

### lncRNAs are differentially regulated during human islet cell death and loss of function

**Figure 3:**
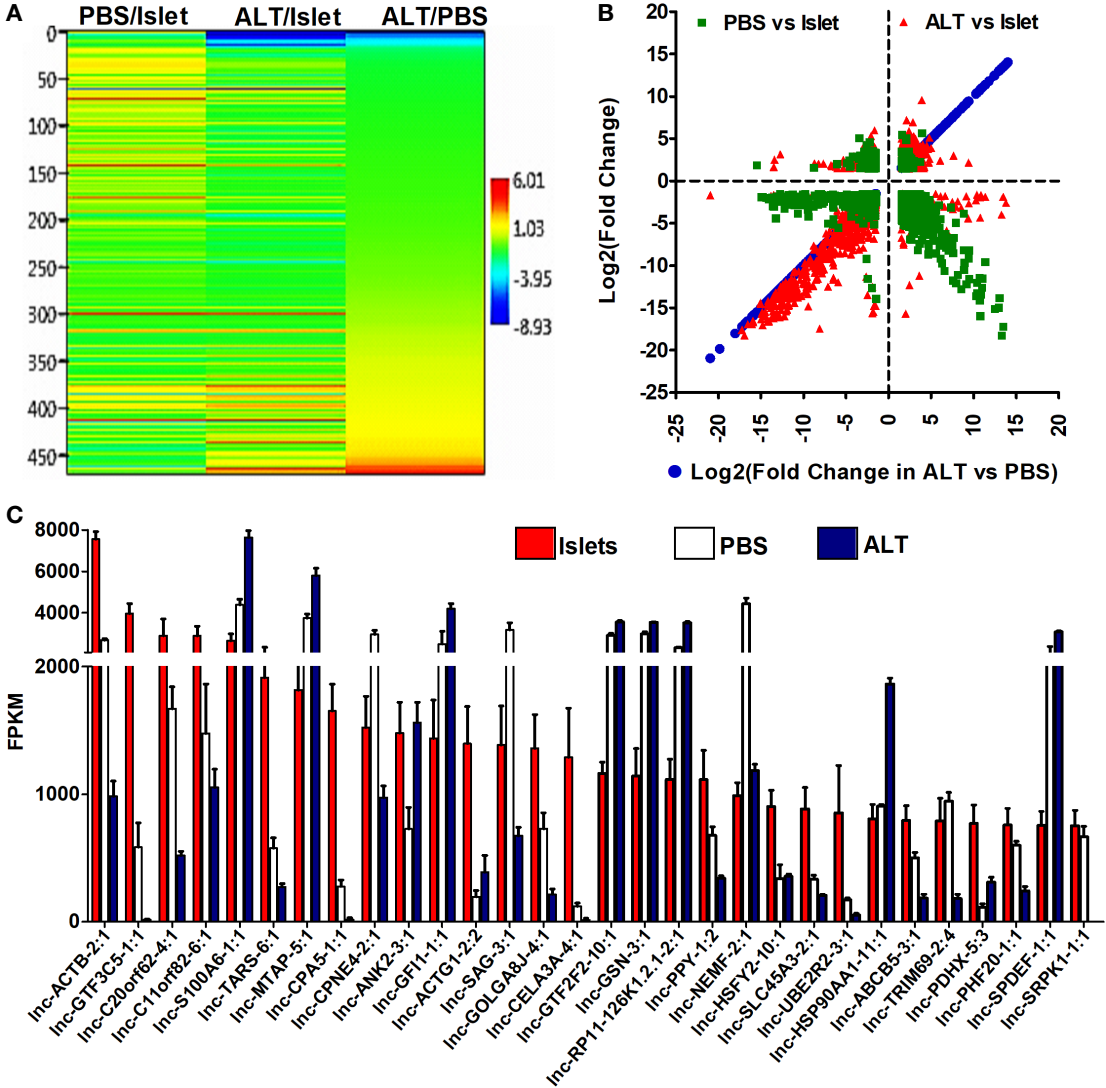
Level and differential expression pattern of lncRNAs in islets and grafts. **(A)** A hierarchical cluster and heatmap of log2(fold change) (*p* < 0.05) of the differentially expressed lncRNAs between ALT-treated islet grafts and PBS-treated islet grafts as well as between grafts and the corresponding islets. **(B)** The log2(fold change) >1.5 with *p* < 0.05 of lncRNAs in ALT vs. PBS were plotted on the *x*-axis and the log2(fold change) in other comparisons are plotted on the *y*-axis to visualize their distribution pattern. **(C)** The pattern and expression level of the top 30 highly islet-enriched lncRNAs in islets, PBS-treated islet grafts, and ALT-treated islet grafts. FPKM: fragments per kilobase per million mapped reads.

Differential expression of lncRNAs in three groups of samples was analyzed to identify their relation to β-cell survival and function. A total of 1,593 lncRNAs were detected in both isolated islets and islet grafts, with or without ALT. Patterns of expression and distribution of these lncRNAs are shown in **Figures 3A, B**. The most aberrant expression of lncRNAs was observed in ALT-treated islet grafts compared to the corresponding islets used for transplantation. A total of 1,093 lncRNAs were differentially expressed in this comparison. Out of 1,093 lncRNAs, 676 were downregulated and 417 were upregulated in ALT-treated islet grafts compared to islets respectively. In PBS-treated islet grafts, 375 were downregulated and 140 were upregulated compared to islets. When comparing the expression of lncRNA transcripts in ALT-treated islet grafts to their PBS-treated counterparts, 857 transcripts were found to be aberrantly expressed. The numbers of upregulated and downregulated lncRNAs were 383 and 474 in ALT-treated islet grafts compared to PBS-treated islet grafts, respectively. Since ALT-treated islet grafts were characterized and validated for their increased β-cell death and decreased insulin secretion compared to PBS-treated islet grafts, these aberrantly expressed lncRNAs might be highly associated with human β-cell survival and functions. All differentially expressed lncRNAs with a *p*-value < 0.05 were taken further into account to extract their neighboring mRNAs, which are also identified as differentially expressed for potential *CIS* regulatory functions. According to the fragments per kilobase per million mapped reads (FPKM), the expression level of all lncRNAs was ranked in islets to select candidate lncRNAs for validation, downstream characterization, and identification of potential functional regulation *in vitro*. The expression of the top 30 islet-enriched lncRNAs in islets and islet grafts with and without ALT are presented in **Figure 3C**. Nearly all lncRNAs fall into two types of expression patterns (**Figure 3C**). Half of the lncRNAs’ expression is very high in islets, medium in PBS-treated islet grafts, and very low in ALT-treated islet grafts (Islet > PBS > ALT). This group of lncRNAs might be associated directly with the loss of human β-cell survival and functions. The rest of the lncRNAs were exhibited in the opposite pattern (Islet < PBS < ALT). This is likely due to the immune response to islet grafts upon the molecular signal exerted by transferred lymphocytes, inducing the subsequent cell death. Expression levels of the 10 lncRNAs identified by sequencing were further validated by real-time qRT-PCR (**Figure S6** in **Supplementary Materials**). The list of differentially regulated lncRNAs with more than ±1.5-fold change in expression in different comparisons, supported by statistically significant expression data, is presented in the Additional results file.

### Identification of *CIS* regulatory transcripts controlled by distinct sets of lncRNAs

**Figure 4:**
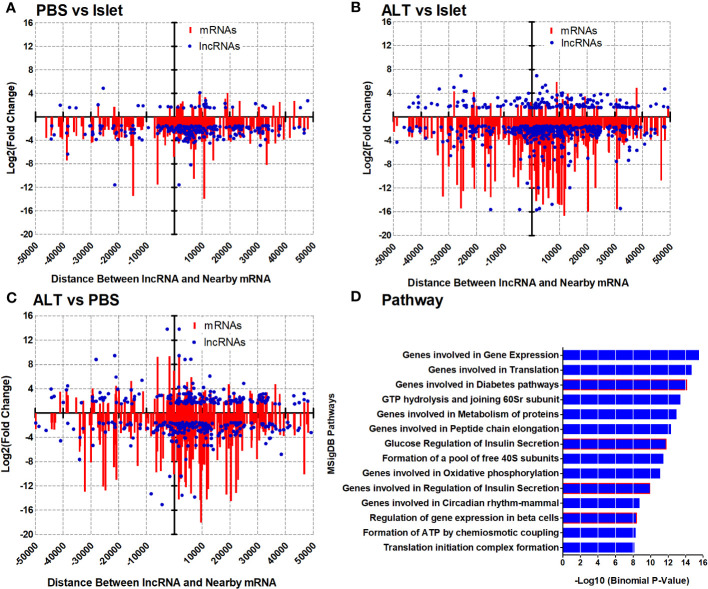
Expression pattern and distribution of closely located mRNA–lncRNAs based on the genomic distance and related pathways. Log2(fold change) of lncRNAs and neighboring mRNAs in PBS vs. islet **(A)**, ALT vs. islet **(B)**, and ALT vs. PBS **(C)** were plotted on the *y*-axis and scattered based on their genomic distance on the *x*-axis. MSigDB Pathways enriched for the differentially expressed mRNAs from closely located mRNA–lncRNA pairs in ALT vs. PBS **(D)**.

A relational analysis of differentially expressed lncRNAs and their potential target mRNAs in the CIS regulatory mechanism was performed by integrating differentially expressed mRNAs along with their genomic coordinates. Since lncRNAs play key roles in regulating the expression of their CIS-associated neighboring or overlapping genes genome-wide, all differentially expressed mRNAs closely located to the differentially expressed lncRNAs were screened out based on their genomic location distributions (within 50 kb) on human chromosomes by using Genomic Regions Enrichment of Annotations Tool (GREAT), and the identified distance between lncRNAs and mRNAs coordinate was validated in the UCSC Genome Browser (UCSC hg19). The cutoff value for the screening was log2 (fold change) > 1.5, *p*-value < 0.05, and a genomic distance of lncRNA isoform from the transcription start site of mRNAs ≤ 50 kb. The distance was calculated using a transcript model instead of a gene model since the distance from mRNA to lncRNA isoforms seems to be more reliable for predicting their CIS regulation (43–45). Altogether, 1,145 mRNA–lncRNA (441 in antisense) pairs were screened out in this way for all three groups of comparisons (PBS vs. Islet, ALT vs. Islet, and ALT vs. PBS). This resulted in extremely complex lncRNA target networks. The analysis of all screened mRNA–lncRNA pairs revealed 236, 532, and 367 mRNA–lncRNA pairs differentially regulated in different relationships in PBS vs. Islet, ALT vs. Islet, and ALT vs. PBS, respectively. In PBS-treated islet grafts compared to islets, 183, 16, and 37 out of 236 matched pairs of mRNAs and lncRNAs were downregulated, upregulated, and oppositely regulated, respectively (Pearson correlation = 0.3899, *p*-value < 0.0001). In the ALT vs. Islet comparison, 378, 19, and 135 out of 532 matched pairs exhibited downregulation, upregulation, and opposite regulation of mRNAs and lncRNAs, respectively (Pearson correlation = 0.3178, *p*-value < 0.0001). Similarly, out of 367 screened pairs of mRNA–lncRNA between ALT-treated islet grafts and PBS-treated islet grafts, 170, 62, and 135 matched pairs of mRNAs and lncRNAs were downregulated, upregulated, and oppositely regulated, respectively (Pearson correlation = 0.4964, *p*-value < 0.0001). Graphical overview of the pattern of expression and distribution of these mRNA–lncRNAs together with corresponding genomic distance is shown in **Figures 4A–C**. The pattern of expression and correlation between the screened differentially expressed mRNAs and lncRNAs indicates that these lncRNAs are potentially involved in the regulation of closely located paired mRNAs in a CIS regulatory mechanism. Non-coordinated pairs of mRNA–lncRNAs may suggest other complex and different regulatory mechanisms across various lncRNAs and their target mRNAs.

### *CIS* regulatory molecules are associated with human β-cell functions and diabetes

Unlike mRNAs, most of the discovered lncRNAs typically lack annotations for their biological functions. We used GREAT analysis to determine the biological meaning of the differentially expressed lncRNAs by exploring the annotations of nearby genes, as screened out differentially expressed matched mRNA–lncRNA pairs, assuming their importance in a *CIS*-regulatory mechanism as mentioned above. Analysis of 1,145 matched mRNA–lncRNA pairs revealed highly enriched GO biological processes, including translation, mRNA metabolic processes, and cellular component disassembly with −log10 (Binomial *p*-value) of 16.9, 15.22, and 12.42, respectively (**Figure S7**; **Table S6** in **Supplementary Materials**). Highly enriched GO cellular components include Ribonucleoprotein complex, Mitochondrial inner membrane, and organelle inner membrane with −log10 (Binomial *p*-value) of 14.92, 12.80, and 12.61, respectively (**Figure S8**; **Table S7** in **Supplementary Materials**). In the case of the associated disease ontology, Pancreatitis with −log10 (Binomial *p*-value) 7.55 was highly enriched for the deregulated mRNAs from the mRNA–lncRNAs pairs (**Figure S9**; **Table S8** in **Supplementary Materials**). The identified common pathways were Gene expression, Translation, Eukaryotic translation initiation, and Insulin synthesis and processing with −log10 (Binomial *p*-value) of 18.70, 14.10, 13.47, and 9.01, respectively (**Figure S10**; **Table S9** in **Supplementary Materials**). Intriguingly, most of the MSigDB Pathways identified as regulated by the aberrantly expressed mRNAs from the selected matched mRNA–lncRNA pairs were highly relevant to β-cell function, survival, and diabetes with −log10 (Binomial *p*-value) > 8.16 (**Figure 4D**; **Table S3** in **Supplementary Materials**). For example, deregulated mRNAs located nearby the differentially regulated lncRNAs were found to be involved in Gene expression, Translation, Diabetes pathways, Metabolism of proteins, Glucose regulation of insulin secretion, Genes involved in the regulation of insulin secretion, and Regulation of gene expression in β cells (**Figure 4D**; **Table S3** in **Supplementary Materials**). Results indicate that differentially expressed lncRNAs might be involved in the regulation of aberrant expression of mRNAs important for β-cell survival, function, and glucose homeostasis. List and number of differentially regulated genes used for the analysis of different pathways, networks, and gene ontologies are presented accordingly in the **Supplementary Data**.

### Spatial expression of islet-enriched lncRNAs across different human tissues

**Figure 5:**
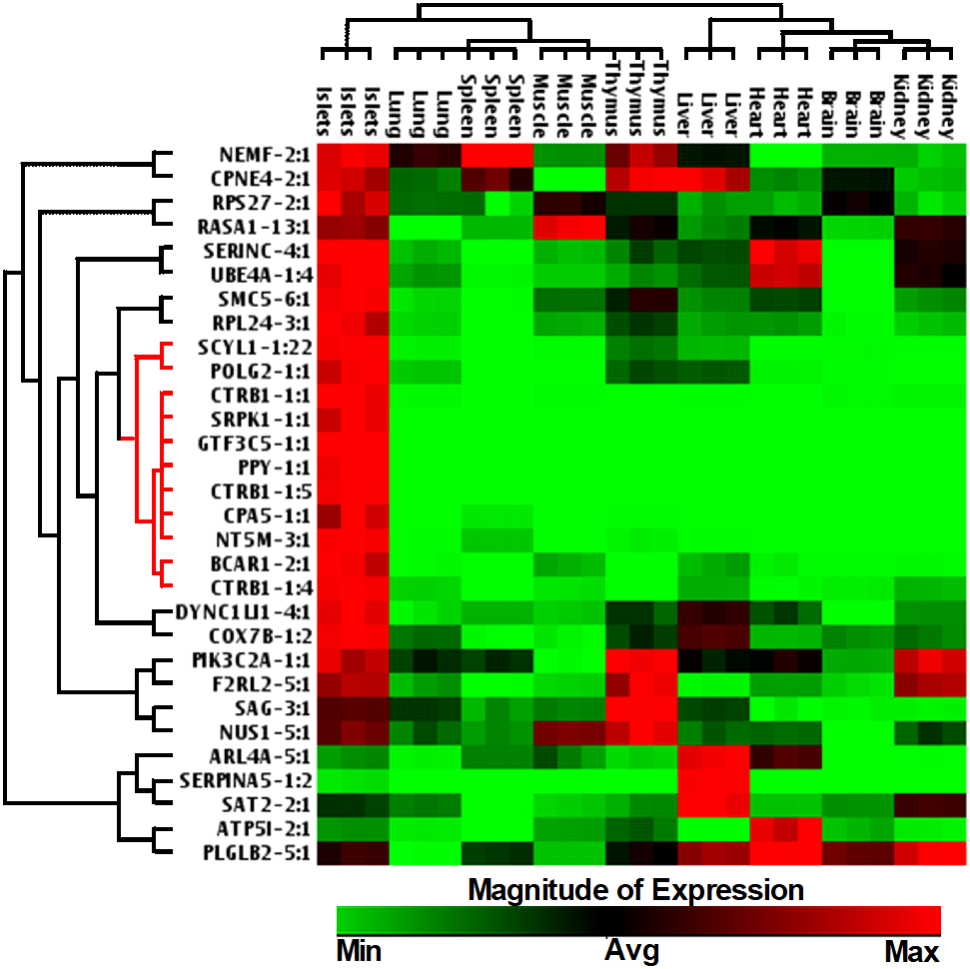
Hierarchically clustered heatmap of the relative expression of selected 30 lncRNAs across different human tissues.

**Figure 6:**
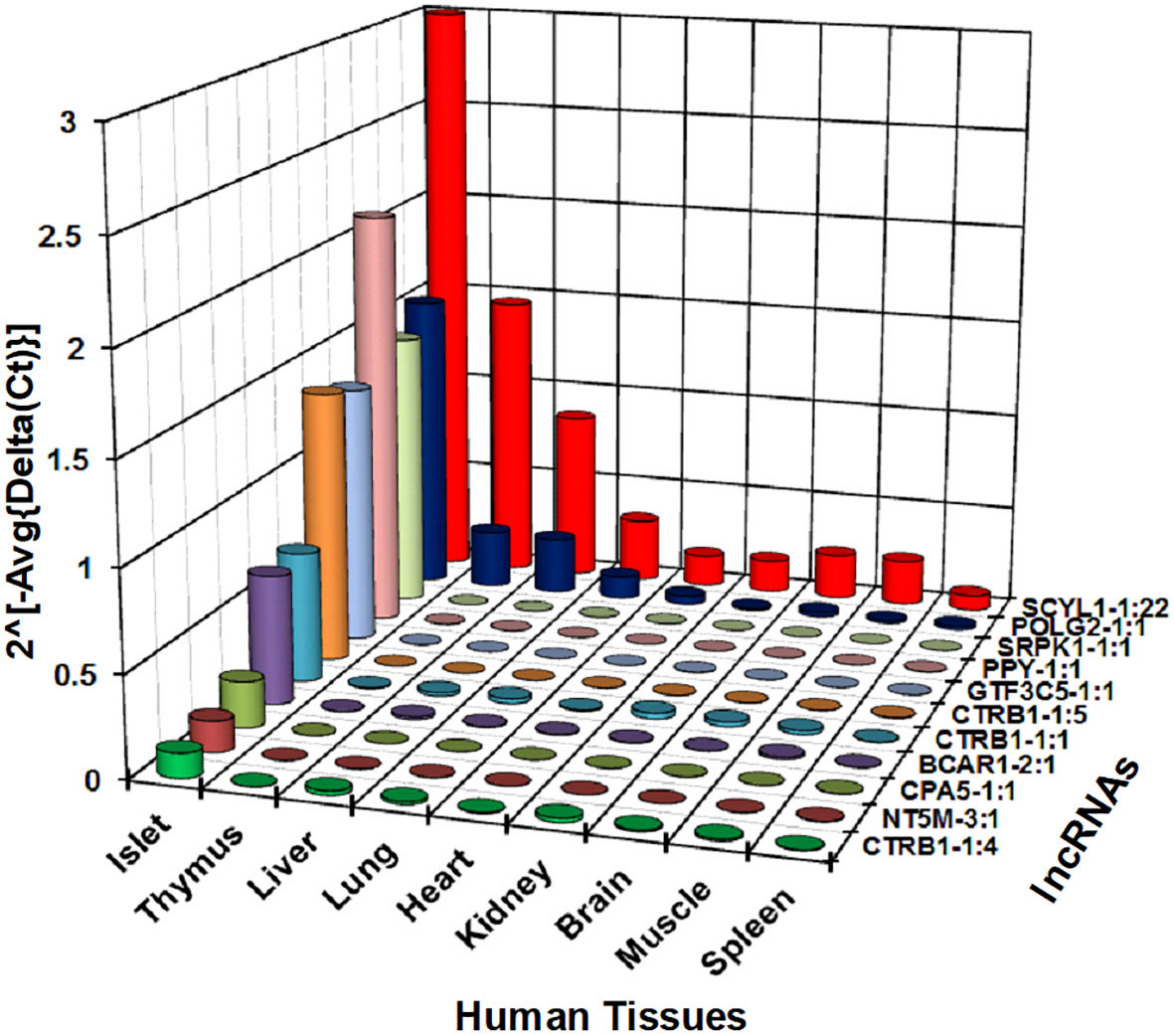
Relative expression level of selected 11 lncRNAs across different human tissues. Expression level of 11 out of the tested 30 lncRNAs shown in **Figure 5** are presented here in a bar diagram. Normalized expression level plotted on the *y*-axis as 2^(−Average(ΔCt))^ value for the lncRNAs across nine different human tissues. Normalization was performed against the mean expression value of human GAPDH and ACTB. Expression of all lncRNAs in human islets was significantly different from all tested tissues (*t*-test, *p* < 0.05).

We next investigated whether the identified lncRNAs have tissue-specific expression profiles, which are ideal as putative biomarkers, or are altered across different human tissues, including pancreatic islets. This suggests that they may have a more common cellular functional role in β-cell biology. We have selected 30 lncRNAs that are highly expressed in islets and differentially regulated between PBS-treated islet grafts and ALT-treated islet grafts, to profile their expression in the human adult heart, liver, lung, kidney, spleen, thymus, muscles, brain, and pooled islets using real-time qRT-PCR (**Figure 5**). A set of lncRNAs was identified as exclusively and highly expressed in human islets. Out of the 30 tested lncRNAs, 26 were highly expressed in islets as seen in the sequencing results. For other tissues, they showed very distinct expression. Most abundant lncRNAs in almost all tissues were NEMF-2:1, CPNE4-2:1, RPS27-2:1, and PLGLB2-5:1. LncRNAs ARL4A-5:1, SERPINA5-1:2, and SAT2-2:1 were found to be expressed only in liver with little or almost no expression in other tested tissues. LncRNAs SERINC-4:1, UBE4A-1:4, and ATP5I-2:1 were found to be heart-enriched, and ATP5I-2:1 had no expression in other tested samples. In addition to islets, lncRNAs that are specific to muscle were RASA1-13:1 and NUS1-5:1. Thymus-enriched lncRNAs were CPNE4-2:1, PIK3C2A-1:1, F2RL2-5:1, SAG-3:1, and NUS1-5:1 (**Figure 5**). This result demonstrates a number of lncRNAs that are specific to a particular tissue and thus may have potential use as tissue-specific biomarkers. There were 11 lncRNAs that were exclusive to islets with no or very low expression in other tested tissues. These include SCYL1-1:22, POLG2-1:1, CTRB1-1:1, SRPK1-1:1, GTF3C5-1:1, PPY-1:1, CTRB1-1:5, CPA5-1:1, NT5M-3:1, BCAR1-2:1, and CTRB1-1:4 (**Figure 6**). An lncRNA with more than double the expression level of the highest level of expression in the nearest human tissue was considered as islet-specific. Accordingly, out of these 11 lncRNAs, 10 were designated as islet-specific lncRNAs (lnc-SCYL1-1:22, POLG2-1:1, CTRB1-1:1, SRPK1-1:1, GTF3C5-1:1, PPY-1:1, CTRB1-1:5, CPA5-1:1, BCAR1-2:1, and CTRB1-1:4) depending on their level of expression in islets compared to other human tissues. These islet-specific lncRNAs hereinafter are denoted as islet-lncRNAs. In addition to nine human tissues we analyzed, expression levels of these 10 islet-lncRNAs were extracted from the expression profile by RNA sequencing of 16 human tissues without islets in the Illumina Human BodyMap2 project that are introduced into ENSEMBL release62 and browse-able in the NONCODEv4 website (**Table 1**). Out of 16 tissues, heart, liver, lung, kidney, and brain were common between our study and the study of the Human BodyMap2 project. Level of expression in islets compared to the level found in the tissue with the highest expression of respective lncRNAs in NONOCODE was 100 to 1,000 times higher (except that SCYL1-1:1 and POLG2-1:1 were more than double in islets compared to heart and thyroid). Although the analysis pipelines are different between the studies, comparing the FPKM value in NONCODE to the quantification of lncRNAs by real-time qRT-PCR in common tissues allowed us to confirm that 10 lncRNAs are human islet-specific. Genomic properties including genomic orientation, location, class, and neighboring genes of these 10 islet-lncRNAs are presented in **Table 1**. Islet-lncRNAs were used for all downstream analysis to characterize them further for the identification of their involvement in human β-cell survival and function.

**Table 1:** Genomic properties and compilation of expression level of islet-lncRNAs from this study and the Human BodyMap Project.

lncRNAs transcripts^#^	Gene ID^#^	Iso^$^	Locus (hg19)	Strand	Class	FPKM in islets	FPKM in PBS islet graft	FPKM in ALT islet graft	N.code* Enriched Tissue (FPKM)	Nearest Gene (Distance to TSS in bp)^
lnc-SCYL1-1:22	lnc-SCYL1-1	23	chr11:65266530-65273915	+	Intergenic	321.526	224.598	820.17	Heart (100.17)	SCYL1 (−22,325), FRMD8 (+116,153)
lnc-POLG2-1:1	lnc-POLG2-1	6	chr17:62499838-62500407	–	Exonic	152.589	34.7796	7.93116	Thyroid (74.41)	POLG2 (−6,969), DDX5 (+2,516)
lnc-CTRB1-1:1	lnc-CTRB1-1	5	chr16:75259972-75262093	+	Intergenic	60.2895	17.1439	1.48629	Kidney (0.069)	CTRB1 (+8,135), BCAR1 (+38,872)
Lnc-SRPK1-1:1	lnc-SRPK1-1	1	chr6:35762887-35765057	–	Exonic	751.402	667.018	0.00328	Testes (4.366)	CLPS (+1,116), CLPSL1 (+15,178)
GTF3C5-1:1	lnc-GTF3C5-1	1	chr9:135945998-135946529	+	Exonic	3,967.02	583.643	13.8299	Testes (22.859)	CEL (+8,899), RALGDS (+50,299)
lnc-PPY-1:1	lnc-PPY-1	2	chr17:42018172-42018882	–	Exonic	202.857	295.959	75.9507	Colon (0.119)	PPY (+1,115), FAM215A(+23,951)
lnc-CTRB1-1:5	lnc-CTRB1-1	5	chr16:75260884-75262093	+	Intergenic	59.2148	8.53556	0.20497	Hela (0.16)	CTRB1 (+8,591), BCAR1 (+38,416)
lnc-CTRB1-1:4	lnc-CTRB1-1	5	chr16:75260415-75261204	+	Intergenic	69.6761	17.1526	0.95125	Testes (0.324)	CTRB1 (+7,912), BCAR1 (+39,095)
lnc-CPA5-1:1	lnc-CPA5-1	1	chr7:130023540-130024902	+	Exonic	1,654.05	276.387	16.1761	Placenta (0.466)	CPA1 (+4,009), CEP41 (+56,857)
lnc-BCAR1-2:1	lnc-BCAR1-2	1	chr16:75238035-75241057	–	Exonic	166.438	48.2731	7.61982	Brain (0.272)	CTRB2 (+1,537), ZFP1 (+57,066)

**^#^** Transcript and Gene ID according to LNCipedia v-4.0.

^$^Number of isoforms.

*lncRNA expression from the RNA sequence expression profile in 16 human tissues from Ensembl release 62: RNA-Seq data from Illumina’s Human BodyMap 2.0 project introduced into Ensembl release 62. Extracted from NONCODEv4. Listed tissue reported to exhibit highest expression (in FPKM) of respective lncRNA.

^Data extracted from “GREAT version 3.00”.

### Species specificity and subcellular localization of islet-lncRNAs

**Figure 7:**
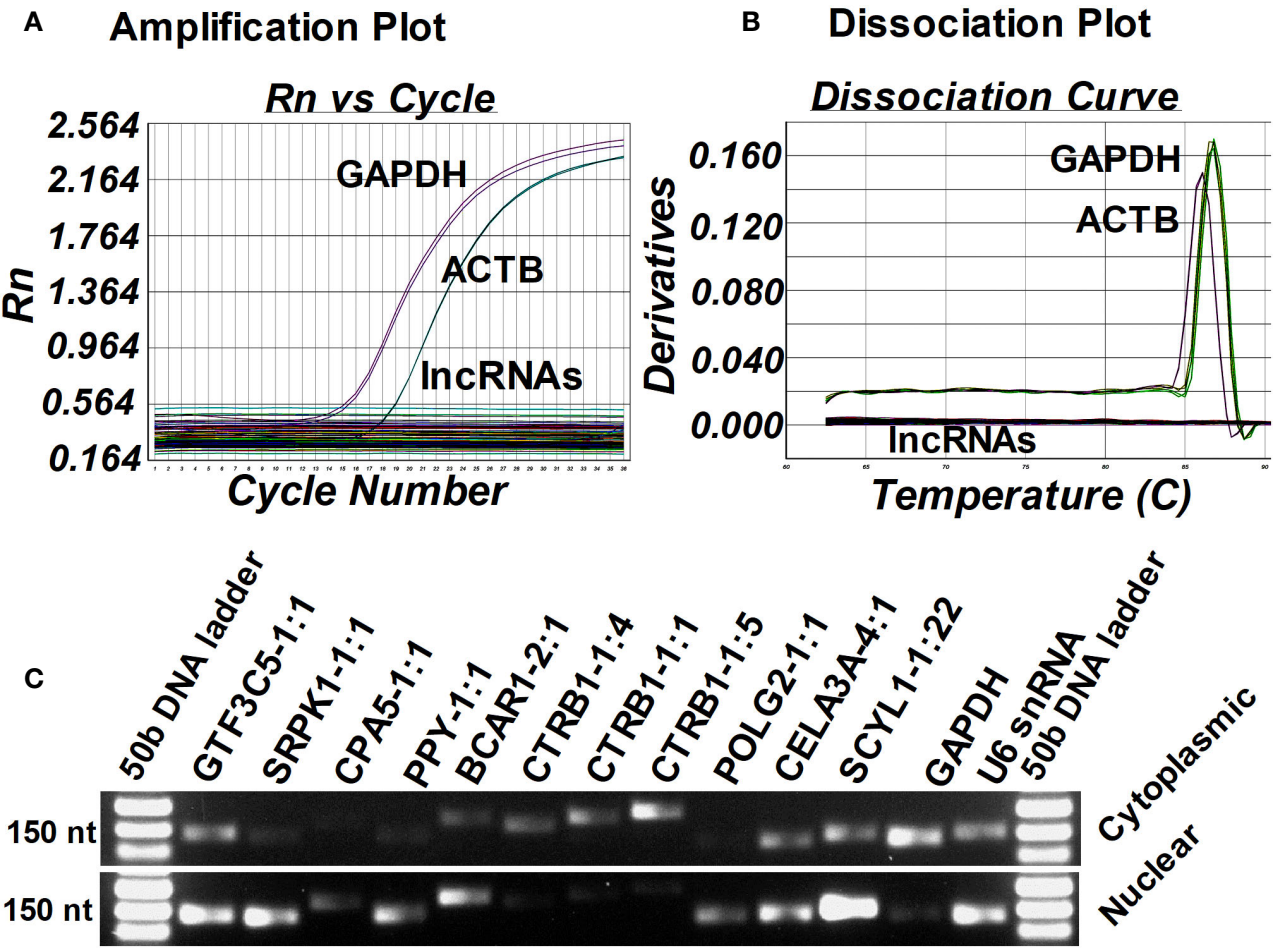
Identification of the source and subcellular location of selected lncRNAs. Amplification plot from ABI 7500 **(A)** and dissociation curve from ABI 7500 **(B)** of selected 10 islet-lncRNAs in the cDNA synthesized from the RNA isolated from the kidney of humanized NOD.scid mice that harbored ALT-treated islet grafts, peripheral blood mononuclear cells (PBMC) isolated from diabetic NOD.scid mice without islet transplantation, and the splenocytes from the diabetic NOD mice that were used for ALT. GAPDH and ACTB were used as endogenous controls. **(C)** Gel electrophoresis image of the amplified candidate lncRNAs in the cDNA synthesized from cytoplasmic and nuclear RNA fractions from the human islets to identify their subcellular localization.

Next, we examined the expression of islet-lncRNAs to check for any contamination with mouse tissue and also tested their conservation status in the mouse genome. Total RNA was isolated from the kidneys of humanized NOD.scid mice that harbored the islet grafts with ALT, PBMCs) isolated from diabetic NOD.scid mice without any islet transplantation, and the splenocytes from the diabetic NOD mice that were used for ALT. Using real-time qRT-PCR, we tested the presence of lnc-SCYL1-1:22, POLG2-1:1, CTRB1-1:1, SRPK1-1:1, GTF3C5-1:1, PPY-1:1, CTRB1-1:5, CPA5-1:1, BCAR1-2:1, and CTRB1-1:4 in mouse kidney, PBMCs, and splenocytes. GAPDH and ACTB were used as endogenous controls. None of the 10 lncRNAs were detected in these mouse tissues (**Figures 7A, B**). This result indicates that the selected lncRNAs originated solely from human islet grafts and not from any other tissues or cells of mouse origin. It also suggests that these lncRNAs are indeed specific to humans. Next, the subcellular location of these lncRNAs was identified by semi-quantitative PCR using the cDNA synthesized from isolated cytoplasmic and nuclear RNA fractions of human islets (**Figure 7C**). GAPDH and U6 RNA were used as reference controls. Lnc-SCYL1-1:22, SRPK1-1:1, CPA5-1:1, PPY-1:1, and POLG2-1:1 were found to be highly expressed in the nuclear fraction of RNA, whereas CTRB1-1:1, CTRB1-1:4, and CTRB1-1:5 were enriched in the cytoplasmic fraction. The remaining lncRNAs were detected in both the cytoplasm and nucleus (**Figure 7C**).

### Selected lncRNA expression in human islets from deceased donors with different BMIs and T2D

**Figure 8:**
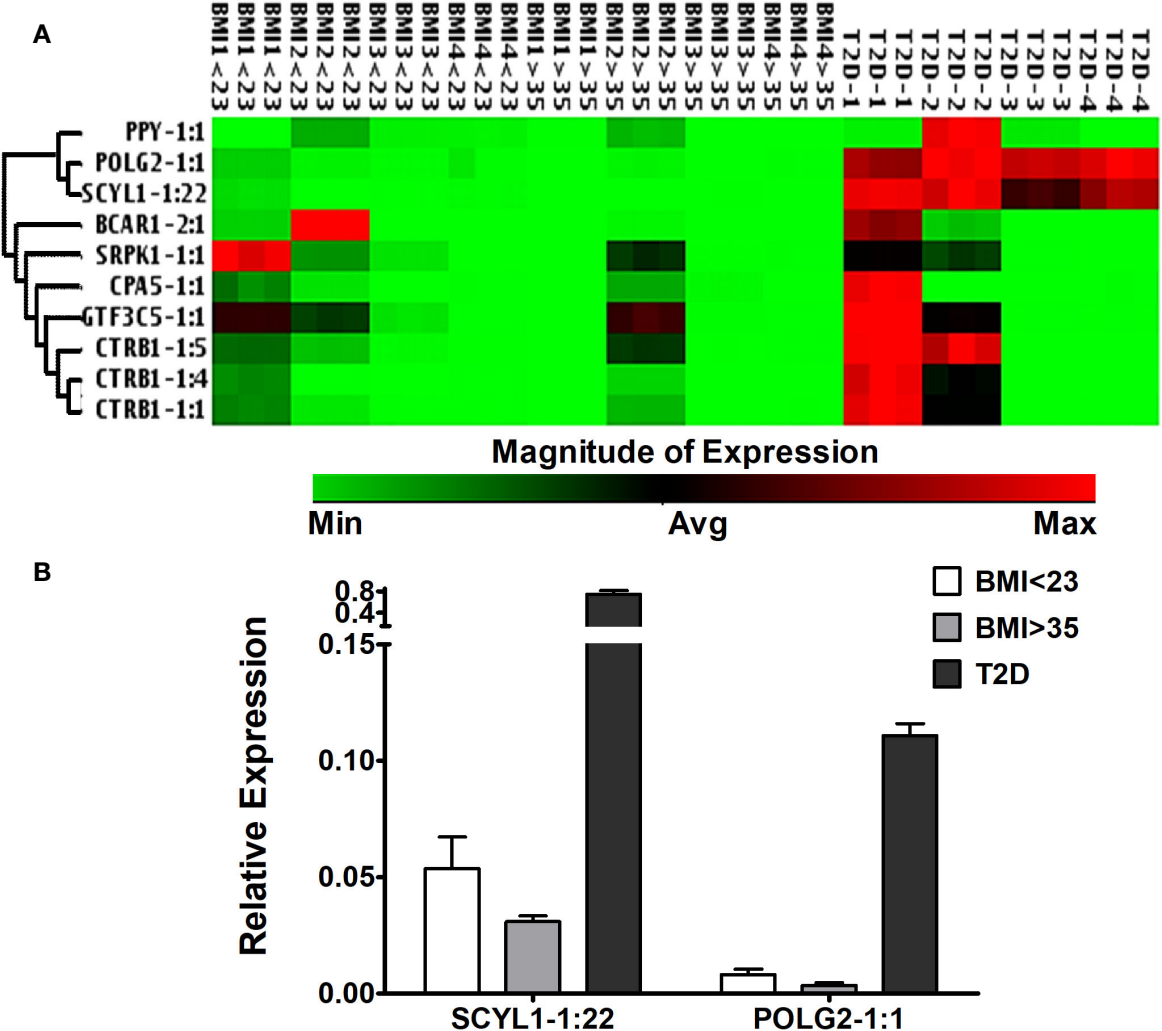
Expression of islet-lncRNAs in the islets from donors with different BMIs and T2D. **(A)** Hierarchically clustered heatmap of the relative expression of islet-lncRNAs in the islets from donors with BMI < 23, BMI > 35, and with T2D (four donors in each group). **(B)** Relative expression of lnc-SCYL1-1:22 and POLG2-1:1 in the islets from donors with BMI < 23, BMI > 35, and with T2D. Expression of lnc-SCYL1-1:22 and POLG2-1:1 was significantly different in all comparisons among the three groups of islets (*t*-test, *p* < 0.05).

Islet-specific lncRNAs, which are differentially expressed in ALT-treated islet grafts compared to PBS-treated islet grafts, were analyzed in the islets from four donors with BMI < 23, four donors with BMI > 35, and four donors with T2D (**Figure 8A**). With the exception of POLG2-1:1 and SCYL1-1:22, most of the lncRNAs were expressed ubiquitously across the tested islets. There was no stable level of expression, even in islets among the donors within the same group. This result indicates that the tested lncRNAs (except POLG2-1:1 and SCYL-1:22) may not be involved in any pathways or functions related to obesity, metabolic disorders, or insulin resistance. Instead, they might be important only for β-cell survival and function, or other cellular functions uncommon among the donors tested. LncRNAs POLG2 and SCYL1 were upregulated in the islets of donors with T2D (**Figure 8B**), and their aberrant expression was observed between islets from the donors with low and high BMI. In addition to islet cell function and survival, these two lncRNAs might be important for cellular function in the islets of donors with T2D and that are also commonly present in islets during loss of β-cell function and survival.

### Regulation of islet-lncRNAs in response to glucose stimulation and cytokine treatment *in vitro*

**Figure 9:**
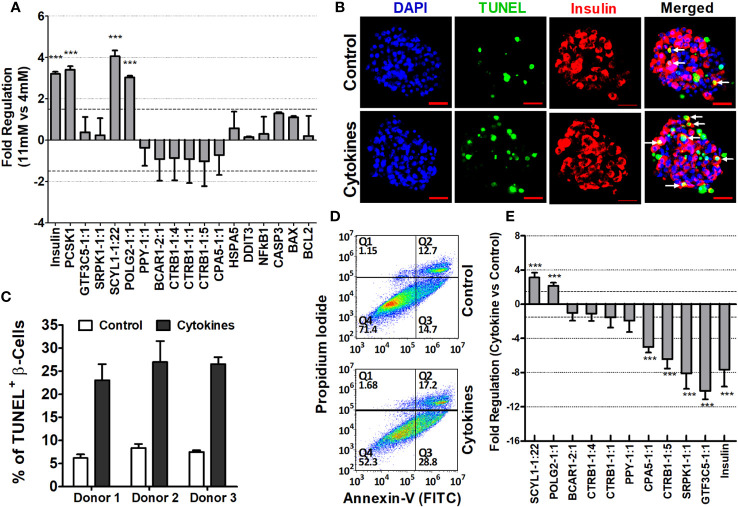
Characterization of islet-lncRNAs in islets cultured with glucose and with and without cytokines. **(A)** Changes in expression of islet-lncRNA, genes related to β-cell secretion (insulin, PCSK1), related to apoptosis (NFKβ1, CASP3, BAX, and BCL2), and related to ER stress (DDIT3 and HSPA5) in response to glucose stimulation. Data presented as mean ± SE. ***Paired *t*-test, *p* < 0.05. **(B)** Images of immunohistochemistry coupled with TUNEL assays on the islets cultured *in vitro* with cytokines (lower panel) and control (upper panel). DNA was stained as blue with DAPI, TUNEL^+^ cells were detected as green, and insulin^+^ cells/β cells were identified red. Apoptotic β cells are indicated as white arrows. **(C)** Percentage of apoptotic TUNEL^+^ β cells in total β cells per islets cultured with and without cytokines from three donors (paired *t*-test, *p* < 0.05). **(D)** Determination of the effect of cytokines on apoptosis of islet cells in flow cytometry assay. Dot plots demonstrating separation of dissociated islet cells into early apoptotic cells in the lower right quadrant (Q3) (Annexin V^+/^PI^-^), necrotic cells in the upper right quadrant (Q2) (Annexin V^+^ and PI^+^), and late apoptotic cells in upper left quadrant (Q1) (Annexin V^-^ and PI^+^). Value in percentage of lower left quadrant (Q1) represents viable cells that were not stained with Annexin-V or PI. **(E)** Fold changes of lncRNAs in the cytokine-treated islets compared to that of control islets. Data presented as mean ± SE. *** Paired *t*-test, *p* < 0.05.

To assess the regulation of islet-lncRNAs in response to glucose-mediated stimulation, human islets were cultured *in vitro* for 60 h in glucose-free RPMI 1640 media supplemented with either 4 mM or 11 mM glucose. Relative expressions of β-cell secretory markers (insulin and PCSK1) were significantly higher in the islets treated with 11 mM glucose compared to those treated with 4 mM glucose. mRNAs related to ER stress (DDIT3 and HSAP5) and related to apoptosis (NFKβ1, CASP3, BAX, and BCL2) showed no significant changes in their expression between the islets treated with different glucose concentrations (**Figure 9A**). In the case of lncRNAs, SCYL1-1:22 and POLG2-1:1 were significantly upregulated in the islets treated with 11 mM glucose compared to those treated with 4 mM glucose. No change in expression was observed for the rest of the lncRNAs under investigation (**Figure 9A**). Thus, differentially regulated islet-lncRNAs might be related to human β-cell physiology under culture conditions. We further determined the expression changes of these islet-lncRNAs in the islets cultured with cytokine-induced apoptosis and inflammation. Culture of islets with cytokine mix (IL-1β, TNF-α, and IFN-γ) is well known for the induction of apoptosis and loss of β-cell function in cultured islets (46). Islets from three donors were cultured *in vitro* independently with and without cytokine mix, and the level of apoptosis for the β cells in particular was assessed by immunostaining of β-cell (insulin) with TUNEL assays simultaneously in the whole islets (**Figures 9B, C**). The percentage of apoptotic β cells per islet was significantly higher in the islets cultured with cytokines (25.48 ± 1.24%) compared to that in the control (7.33 ± 0.63, *p* = 0.0013). Additionally, the level of apoptosis in the dissociated islet cells after culturing was detected using flow cytometry (**Figure 9D**). Real-time qRT-PCR was performed to detect the relative expression of lncRNAs in the islets cultured with and without cytokine mix. Most of the tested islet-lncRNAs were deregulated in the islets in response to cytokines, as observed similarly in the ALT-treated islet grafts compared to PBS-treated islet grafts. The fold regulation (negative inverse of fold change) of the expression of these lncRNAs in the pooled data from three donors is shown in **Figure 9E**. LncRNAs SCYL1-1:22 and POLG2-1:1 were upregulated in the cytokine-treated islets compared to control from all three donors. The remaining islet-lncRNAs were downregulated in the cytokine-treated islets across all donors. LncRNAs that are significantly decreased in the cytokine-treated islets might be regulated differently in relation to the difference in stimuli and response across the donors against cytokines.

### Islet-lncRNAs are secreted in exosomes of human islets and differentially regulated after cytokine treatment *in vitro*

**Figure 10:**
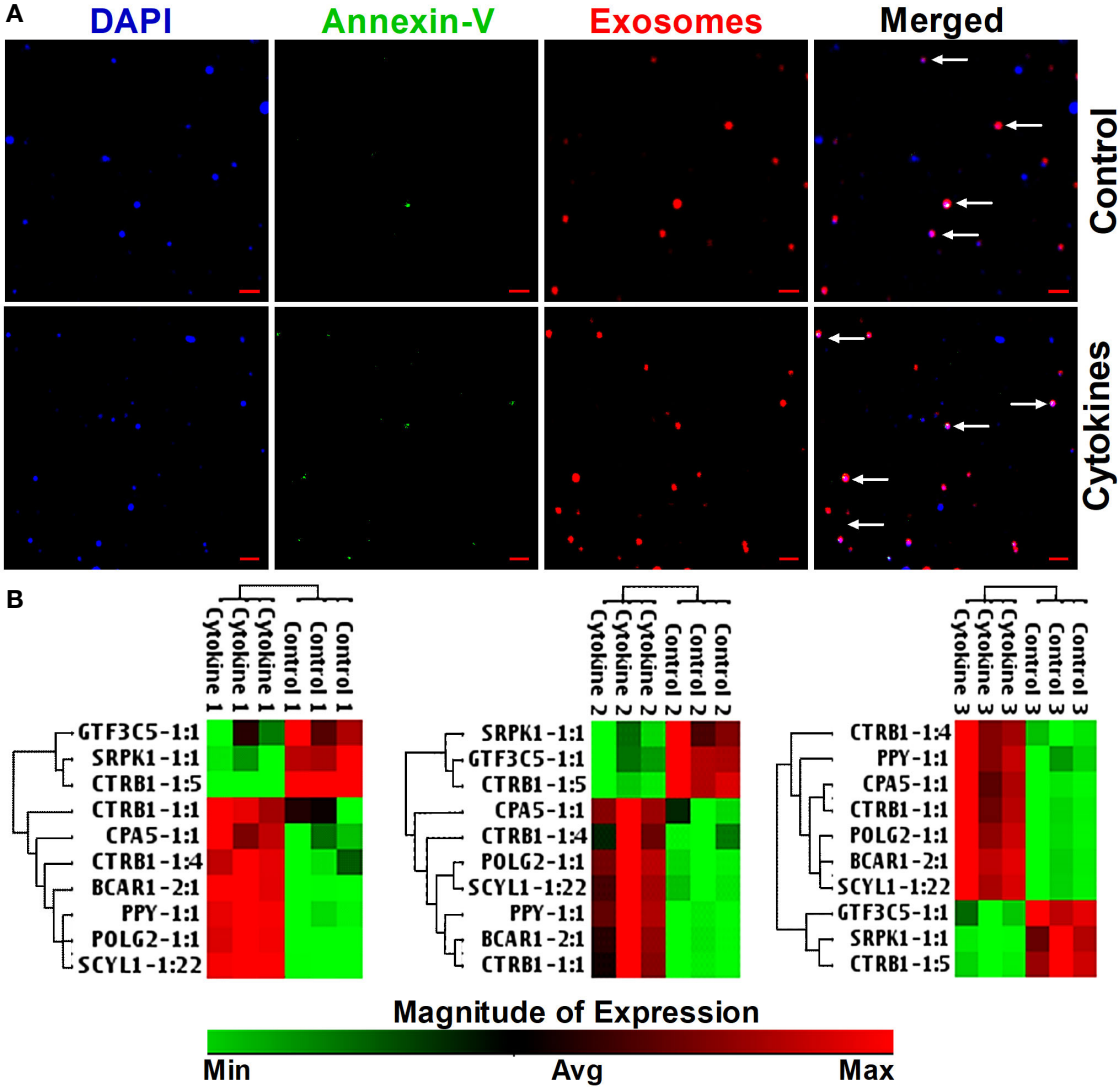
Staining of exosomes and detection of lncRNA in exosomes in response to cytokines. **(A)** Images of exosomes precipitated from culture media of cytokine-treated islets (lower panel) and from control islets (upper panel). DNA was stained as blue with DAPI, Annexin-V was detected as green, and the phospholipid bilayer membrane of exosomes was stained as red. Scale bars represent 1 µm. **(B)** A hierarchically clustered heatmap of the relative expression of islet-lncRNAs in the exosomes precipitated from islets (from three donors) cultured with and without cytokines. Treatment numbers 1, 2, and 3 represent the islets from donors 1, 2, and 3, respectively.

**Figure 11:**
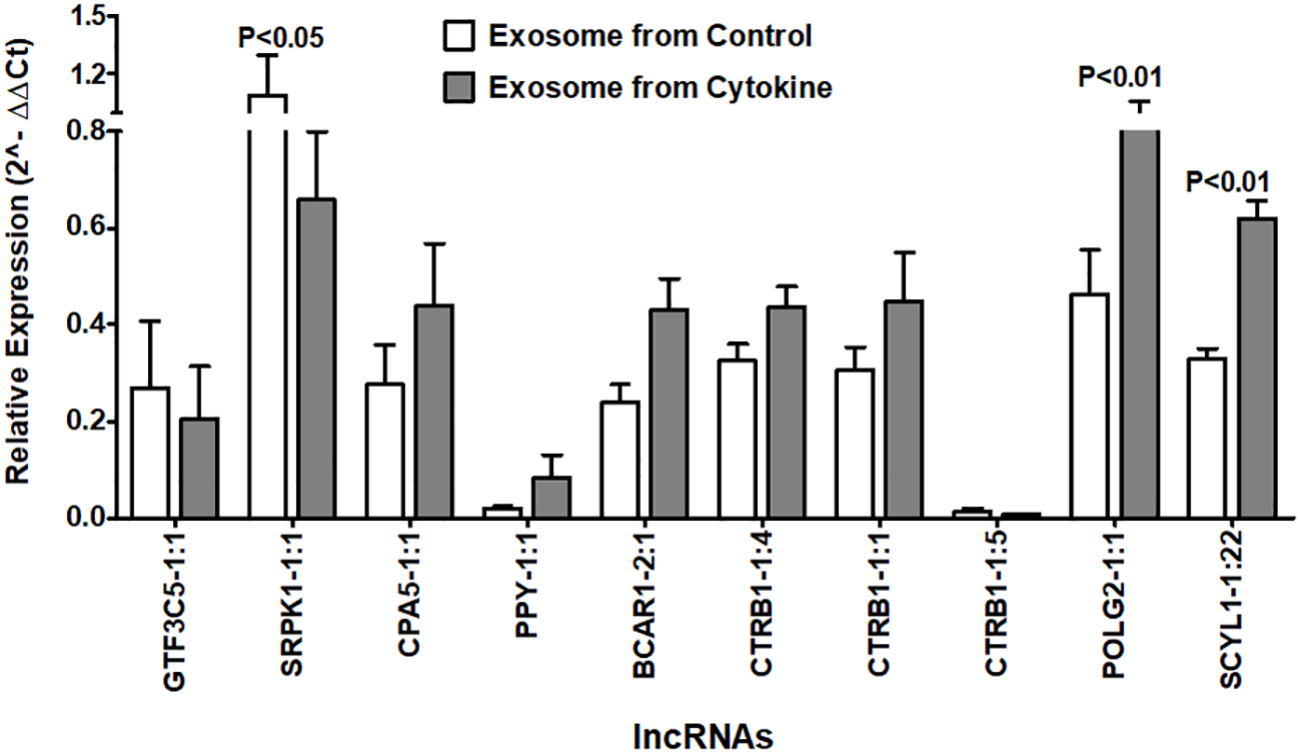
Relative expression of islet-lncRNAs in the exosome precipitated from culture media of cytokine-treated islets and from control islets. Normalized expression level of selected islet-lncRNAs plotted on *y*-axis as 2^(−Average(ΔCt))^ value for the lncRNAs in two groups of exosomes. Normalization was performed against the expression value of human GAPDH. Expression of all lncRNAs (except GTF3C5-1:1) was significantly different in exosomes of cytokine-treated islet culture media compared to that of the control (*t*-test, *p* < 0.05).

Next, we investigated whether the identified islet-specific lncRNAs, which are differentially regulated during the loss of islet cell function and survival in a humanized mice model and in the islets cultured with cytokines, can be detected in the exosomes from the islet culture media. Exosomes are well known to contain DNA and to be enriched with phosphatidylserine in the lipid bilayer facing the extracellular environment (47–49) with a high affinity to bind Annexin-V (50). We were able to visualize the purified exosomes stained with DAPI for DNA, FITC-Annexin-V for phosphatidylserine, and Vybrant DiI for the lipid bilayer in laser scanning confocal microscope (**Figure 10A**). All 10 of the tested lncRNAs were detected in the exosomes by real-time qRT-PCR (**Figure 10B**). Interestingly, some lncRNAs showed similar patterns to those found in both cultured islets and ALT-treated islet grafts. LncRNAs GTF3C5-1:1, SRPK1-1:1, and CTRB1-1:5 were found to be significantly downregulated in the exosomes from the media of cytokine-treated islets compared to their control counterpart (*p* < 0.05). LncRNAs SCYL1-1:22 and POLG2-1:1 were upregulated in the exosomes of the cytokine-treated islets compared to the control islets (**Figure 11**, *p* < 0.05). The pattern of expression of these six lncRNAs was similar across all three donors (**Figure 10B**, except GTF3C5-1:1 in donor 1, *p* > 0.05). This expression pattern was observed in both cytokine-treaded islets *in vitro* and ALT-treated islet grafts in humanized mice. Expression of the remaining tested lncRNAs was higher in the exosomes from the media of cytokine-treated islets compared to control. The presence and differential regulation of islet-specific lncRNAs in the exosomes suggest that they might be involved in the cellular cross-talk between β cells under different physiological conditions, related to their functional status during culture conditions or the process of apoptosis. These lncRNAs could also communicate through exosomal shuttles to other cell types within the islets in a paracrine manner.

## Discussion

Although lncRNA-mediated posttranscriptional and epigenetic landscapes of gene regulation have been found to be related to many human diseases and modifications in cellular function (12–15), the regulation governing human β-cell function and survival is not well known. Several recent studies explore the influence of lncRNAs on pancreatic cell function. These investigations encompass a range of topics, including the role of pancreatic lncRNAs like Paupar in regulating glucagon secretion by α-cell genes in mice (29), the participation of lncRNAs in shaping transcriptional networks and chromatin structure within human pancreatic β cells (30), and the significance of lncRNAs such as HASTER, which acts as an antisense regulator of HNF1A, in maintaining HNF1A concentrations in pancreatic β cells during the development of diabetes mellitus (31). The present study explored the association of lncRNAs with human pancreatic islets under induced apoptosis through adoptive lymphocyte transfer in the humanized mice. Humanized mice with transplanted functional human islets were generated, and subsequently, ALT was performed using diabetic NOD mice to induce β-cell death in human islet grafts. Morphometric and molecular characterization of islet grafts treated with ALT and their control counterparts (PBS) validated the islet cell death in the ALT-treated islet grafts. In addition, significantly higher apoptosis and ER stress were confirmed by the quantification of related genes. Through whole transcriptome sequencing, deregulated mRNAs and lncRNAs due to loss of β-cell function and survival were identified. For mRNA analysis, all annotations and analyses were done using the gene model according to the Ensembl release 75. We analyzed the lncRNAs in their isoform level since most of the lncRNAs have multiple isoforms that are differentially regulated. It has been identified that more than 25% of lncRNA genes show evidence of alternative splicing with at least two different transcript isoforms per gene locus (51). Different isoforms from the same lncRNAs were found to have different expression patterns in the analyzed sample. This renders their analysis as gene model for downstream analysis and validation, providing more challenges. It has been shown that the analysis of expression at the isoform level provides more prognostic stratification and classifies better molecular signatures for biomarker discovery than gene-level expression profiles (43–45). Until now, LNCipedia and NONECODE are the only databases that provide annotation of lncRNAs at their isoform level. Thus, for lncRNAs, we used transcript model according to LNCipedia version 3.0, which enabled us to analyze the variation or alteration of lncRNAs at their isoform level. In addition, this analysis allowed us to distinguish the isoforms and analyze them individually, which are generated due to natural transcriptomic dynamics in the islet grafts caused by induced β-cell death in the islet grafts due to ALT.

Analysis of sequencing reads revealed aberrant expression of both mRNAs and lncRNAs in the ALT-treated islet grafts, compared to PBS-treated islet grafts (**Figures 2**, **3**). Differentially expressed mRNAs in any of the sample dynamics were found to be involved in several pathways related to “Death receptor signaling, Endoplasmic reticulum stress pathway, ERK/MAPK, ERK5, IL-8, ILK, iNOS, Jak/Stat, p38 MAPK, Pancreatic Adenocarcinoma signaling, and MIF regulation of innate immunity pathways” (**Figure 2B**; **Table S1** in **Supplementary Materials**). The most affected disease and functional regulations by these aberrantly expressed mRNAs was “Cell death and survival, and Cell cycle regulation” (**Figure 2C**; **Table S2** in **Supplementary Materials**). Aberrantly expressed mRNAs between ALT-treated islet grafts and PBS-treated islet grafts revealed the canonical pathways involved in cell death and survival, autoimmunity, and T1D signaling pathways (**Figures 2B, C**, **Tables S1, S2** in **Supplementary Materials**). All these signaling pathways and regulation are already evident as major regulators of β-cell death and are known to be important in diabetes, including both T1D and T2D (52–55). The result obtained from the pathway analysis of differentially expressed mRNAs provides functional validation of loss of human β-cell survival and functions in ALT-treated islet grafts and suggests that the aberrantly expressed mRNA transcripts are involved in the cellular changes related to the loss of β-cell survival and function.

The results obtained from the pathway analysis of differentially expressed mRNAs provide the functional validation for the loss of human β-cell survival and functions in ALT-treated islet grafts. It suggests that the aberrantly expressed mRNA transcripts are involved in the cellular changes resulting from the loss of β-cell survival and function. The expression of lncRNA transcripts in the ALT-treated islet grafts showed that a number of lncRNAs are deregulated when compared to the PBS-treated islet grafts (**Figure 3A**). A small-scale analysis of expression of a few lncRNAs (KCNQ1OT1 and HI-LNC45) was previously reported to be differentially regulated in the islets from T2D donors compared to those in non-diabetic donors (5). LncRNA ANRIL was differentially expressed in β-cell dysfunction *in vitro* (26). An analysis of lncRNA expression in the mouse MIN6 cell line in response to cytokines *in vitro*, as well as in diabetic NOD mice, identified a handful of differentially expressed lncRNAs (25, 56). However, most of the identified lncRNAs are absent in the human genome, and hence, it is difficult to correlate their functional involvement in human β-cell death and functions. Until now, the large-scale discovery of lncRNA-mediated regulation of either human β-cell apoptosis or β-cell death in T1D patients has not yet been studied. For the first time, we identified the changes in lncRNAs in response to induced human pancreatic islet cell death and loss of function (**Figures 3B, C**). The level of lncRNA expression in ALT-treated islet grafts with induced human islet cell death and loss of function, compared to their PBS-treated counterpart, revealed aberrantly expressed transcripts (**Figure 3A**). These aberrantly expressed lncRNAs might be highly associated with the loss of human islet cell survival and functions. These lncRNAs represent potential candidates for studying their modulation during the development of T1D. CIS-regulatory gene expression by the lncRNA has been previously observed in studies such as the one involving the lncRNA HASTER, which was shown to play a critical role in maintaining the stability of its target gene, HNF1A. This function of HASTER ensures the precision of cell-specific transcription factor programs, uncovering a distinct regulatory element that impacts transcription and leads to the onset of diabetes mellitus when disrupted (31).

Unlike coding genes, the functions of lncRNAs are largely unknown. A simple way to predict the putative functions of lncRNAs is through the construction of co-expression networks involving coding and lncRNAs (57). The analysis of screened 236, 532, and 367 differentially regulated mRNA–lncRNA pairs (**Figures 4A–C**) in various comparisons (PBS vs. Islet, ALT vs. Islet and ALT vs. PBS) identified common pathways, including insulin synthesis and processing (**Figure 4D**; **Table S3** in **Supplementary Materials**). The MSigDB Pathways identified as being regulated by the aberrantly expressed mRNAs from the selected matched mRNA–lncRNA pairs include diabetes pathways, metabolism of proteins, glucose regulation of insulin secretion, regulation of insulin secretion, and regulation of gene expression in β cells (**Figure 4D**; **Table S3** in **Supplementary Materials**). Differentially expressed lncRNAs were found to have a positive correlation with the differentially regulated mRNAs. Altogether, our data imply that differentially expressed lncRNAs might be involved in the regulation of neighboring mRNAs through a CIS regulatory mechanism in processes related to islet cell death.

Most of the lncRNAs are expressed in a tissue- or cell-specific manner compared to mRNA genes (10, 11). Considering the higher level of expression in islets, 30 lncRNA isoforms (at least one isoform among all the isoforms detected for each highly islet-enriched lncRNAs) were characterized for their spatial expression pattern across different human tissues (**Figures 3C**, **5A**). This allows us to screen the distribution of islet-enriched lncRNA expression in other tissues to characterize them for general cellular function or to screen islet-specific lncRNAs that are involved particularly in islet biology. This could be used for further characterization as well as projected as potential biomarkers. At least 18 islet-enriched lncRNAs were found to be also highly expressed in one or more other tested tissues (**Figure 5**). Even though they were enriched in islets, many of them from our top list were not islet-specific. These lncRNAs might be involved in the general cellular processes rather than β-cell-specific function and other regulatory action required by all lncRNA-enriched tissues. The rest of the lncRNAs were found to be enriched and specifically expressed in islets. Among them, the expression level of eight lncRNAs consisting of 10 isoforms (SCYL1-1:22, POLG2-1:1, CTRB1-1:1, SRPK1-1:1, GTF3C5-1:1, PPY-1:1, CTRB1-1:5, CPA5-1:1, BCAR1-2:1, and CTRB1-1:4) were found to be more than double in human islets compared to any other tested human tissues (**Figure 6**). We checked the RNA-Seq data on 16 human tissues where pancreas or islets were not studied from Illumina’s Human BodyMap 2.0 project introduced in Ensembl release 62 online through NONCODEv4 (**Table 1**). Comparing the FPKM value for our 10 lncRNA isoforms based on our RNA-Seq result in islets, the FPKM value from NONCODEv4 and quantification through RT-PCR in common tissues of our study has confirmed their islet specificity (**Table 1**). Some of them are detected as being 1,000 times higher in islets compared to the highest level of expression in the enriched tissues shown in NONCODEv4 (58).

Expression levels of previously identified islet-enriched lncRNAs (5) were found to be below the level of our selected 30 lncRNAs (**Figure 5**). For example, HI-LNC25 (lnc-DHX35-6), HI-LNC12 (lnc-TSPAN17-1), HI LNC72 (lnc-CA8-7), HI-LNC60 (lnc-KCNV2-9), HI-LNC80 (lnc-SCD-1:15), HI-LNC79 (lnc-TBCEL-2:1), HI LNC70 (lnc-CIT-8:1), HI-LNC78 (lnc-DICER1-1:1), and HI-LNC75 (lnc-KCNJ16-1) were also found to be higher in the islet of our study, but their total read counts (FPKM) were less than those of our selected lncRNAs and were found not to be differentially regulated in our study. Since the 10 identified lncRNA transcripts in our study are highly islet enriched (**Figure 6**), islet-specific, and also differentially regulated in the ALT-treated islet grafts compared to PBS-treated islet grafts, they are not common to the earlier study (5). We tried to compare all the islet-enriched lncRNAs to α- and β-cell-specific lncRNAs proposed in another study (27), where they presented only the genomic coordinates in the supplementary information without any annotations. Because of the lack of annotation for those lncRNAs in any databases, through the genomic region BLAST, we found none of them were matched to the genomic region of any of our islet-enriched lncRNAs. Identified islet-lncRNAs in this study could hence be considered as therapeutic targets and potential biomarkers for the loss of human β-survival and functions, if they are not specific to other tissues that were not investigated in this study.

It has been shown that lncRNAs are less conserved across species compared to protein-coding genes (9), and notable species differences in lncRNAs related to islets between mice and human were observed (59). Through wet-lab experiments, the expression of our islet-lncRNAs was tested in mouse tissue, but none of them were found to be present (**Figures 7A, B**). This ensures that none of the islet-lncRNAs were from mouse genome due to contamination with islet grafts. No orthologous sequence for the islet-lncRNAs was found to be present in mouse genome; thus, they are not conserved in mouse. This supports the notion that tissue-specific lncRNAs are less conserved across species. In the previous study, only 2 out of 145 mouse lncRNAs expressed in mouse islets were shown to have a human orthologous sequence (5).

In addition to the expression of lncRNAs in the islet grafts with a loss of β-cell survival, the regulation of islet-lncRNAs was studied in the islets from donors with different BMIs and T2D (**Figure 8A**), as well as islets stimulated with different glucose concentrations to explore their metabolic pathways and islet cell biology (**Figure 9A**). Many of the lncRNAs have previously been shown to be deregulated in the islets of donors with T2D (5), but whether they are deregulated in donors with different BMI is unknown. We identified two islet-lncRNAs (SCYL1-1:22 and POLG2-1:1) that were significantly upregulated in the islets with T2D across all tested donors, while none of the other islet-lncRNAs were found to be consistently deregulated in the islets with different BMIs (<23, >35) and T2D across the donors (**Figure 8B**). Testing islets from more donors might be necessary to obtain a clearer picture of the regulation of islet-lncRNAs in relation to metabolic pathways. Interestingly, the expression of the mentioned two islet-lncRNAs was found to be similarly affected by the glucose stimulation (**Figure 9A**). Islet cells exhibit a transcriptional response of both coding and non-coding genes to increased demand under glucose stimulation in mouse (59) and rat (60), and glucose-mediated β-cell failure as well as stress response in T2D has been reported (61). LncRNAs have been shown to be moderately induced in response to high glucose concentration (5). A study by Moran et al. (5) has shown consistent upregulation of two lncRNAs (HI-LNC78 and HI-LNC80) in a glucose-dependent manner. The human ortholog of the mouse lncRNA blinc3 was found to exhibit differential expression in the islets of patients with T2D, and this expression was linked to the donors’ BMI (23). Deletion of βlinc1 has been shown to impair islet development and disrupt glucose homeostasis in adult mice (62). A PCR-based analysis demonstrated higher expression of Meg3 in Balb/c mouse islets compared to other exocrine glands. Furthermore, Meg3 expression decreased in islets of T1DM (NOD female mice) and T2DM (db/db mice) models. In addition, the expression of Meg3 in Min6 cells and isolated mouse islets was found to be dynamically regulated by glucose levels (63). A particular lncRNA known as βFaar has been identified as highly expressed in mouse islets. However, studies have shown that βFaar expression is significantly decreased in the islets of obese mice. This reduction in expression has led researchers to propose that βFaar plays a critical role in the development of obesity-induced β-cell injury and apoptosis (64). In our study, we found no human orthologs for lncRNA blinc3, HI-LNC, Meg3, and βFaar that were differentially expressed. In this study, human islets cultured *in vitro* under appropriate conditions for 60 h in the presence of low (4 mM) and high (11 mM) glucose were found to have an induction of transcripts related to β-cell function (Insulin) and secretion (PCSK1) (**Figure 9A**). We found that 2 out of the 10 tested lncRNAs (SCYL1-1:22 and POLG2-1:1) were significantly upregulated in the islets exposed to high glucose compared to those exposed to low glucose (**Figure 9A**), while no significant difference was observed in the expression of apoptosis and ER stress marker genes between high-glucose-treated and low-glucose-treated islets (**Figure 9A**). This confirms that either the low response of the remaining unregulated lncRNAs is deregulated only during β-cell apoptosis or the period of culture (60 h)/glucose level was not ideal to change their expression. A notable difference was observed in the expression of the same islet-lncRNAs in human islets cultured with cytokines (**Figure 9E**). Four islet-lncRNAs (CPA5-1:1, CTRB1-1:5, SRPK1-1:1, and GTF3C5-1:1), which were found to be unregulated upon glucose stimulation, are significantly downregulated in islets with β-cell apoptosis induced by cytokine treatment compared to the control counterpart (**Figure 9E**).

We found that the lncRNA SCYL1-1:22 and POLG2-1:1 are upregulated in islets treated with cytokines, in islets with T2D, and in islets subjected to glucose stimulation (**Figures 8B**, **9A, E**). It should be mentioned that SCYL1-1:22 was significantly upregulated in the ALT-treated islet grafts compared to PBS-treated islet grafts. These results imply that these two RNAs are important either for glucose homeostasis through metabolic pathways or for β-cell apoptosis. In addition to ALT-treated islet grafts and cytokine-treated islets, the expression of SCYL1-1:22 and POLG2-1:1 was widely secreted and upregulated in exosomes isolated from the media of *in vitro* cultured islets undergoing cytokine-induced apoptosis (**Figures 10B**, **11**). The predicted role of lncRNA SCYL1-1 has been shown in NONCODEv4 to be associated with the regulation of glycogen biosynthetic process. Patients with T2D and individuals at risk for the disease display impaired insulin-stimulated glycogen synthesis along with insulin resistance (65, 66). The protein coding gene nearest to SCYL1-1:22 is SCYL1, a protein kinase involved in the regulation of transcript and retrograde vesicle-mediated transport [UniProtKB - Q96KG9 (NTKL_HUMAN)]. POLG2-1:1 is located 6,969 bp antisense to a nuclear DNA polymerase gamma 2, which is known to be associated with mitochondrial DNA (mtDNA) replication and eventually mtDNA depletion. Changes and mutations of POLG2 have been correlated with mitochondrial-associated metabolic disorders, including T2D (67).

The expression of lncRNA CPA5-1:1, CTRB1-1:5, SRPK1-1:1, and GTF3C5-1:1 was downregulated and showed a similar pattern both in ALT-treated islet grafts compared to PBS-treated islet grafts and in islets treated with cytokines compared to their respective control counterpart. The peptidase encoding genes CPA1, CTRB1, and CLPS and the lipase encoding gene CEL are found to be closely located to the lncRNAs CPA5-1:1, CTRB1-1:5, SRPK1-1:1, and GTF3C5-1:1 respectively. Interestingly, all of these have been shown to have significant regulatory roles in diabetes. CTRB1, which encodes chymotrypsinogen via incretin pathway, has been shown to be associated with the development of diabetes and response to DPP-4 inhibitor treatment (68). CPA1, a pancreas-enriched carboxypeptidase A, is a family of zinc metalloproteases that preferentially cleaves C-terminal branched-chain and aromatic amino acids from dietary proteins. It is strongly associated with early-onset chronic pancreatitis (69). The gene near SRPK1-1:1 is GLPS, a cofactor required by pancreatic lipase for efficient dietary lipid hydrolysis. It has also been shown to be associated with insulin secretory function in non-diabetic humans and is suggested as a novel candidate gene for the development of T2D (70). Mutations in carboxyl ester lipase (CEL) has been shown to cause a syndrome of diabetes and pancreatic exocrine dysfunction (71). Therefore, the regulation of islet-lncRNAs in human islets with induced islet cell death and loss of function, either by ALT or by cytokines, as well as in the exosomes secreted by islets suggests that these lncRNAs could be involved in the regulation of such genes important in diabetes.

Collectively, it appears that the repertoire of islet-lncRNAs is dynamically regulated under islet graft *in vivo* and islet *in vitro* conditions of apoptosis, T2D, and glucose stimulation. Furthermore, they can be detected in the exosome secreted from the islet. Because of the low detection efficiency of islet-lncRNAs as free circulating RNAs in the plasma or serum sample, an exosome-oriented study from the patient serum or plasma would be a more promising approach for leading biomarker discovery in human β-cell function and survival.

## Conclusion

In this study, through sequence-based transcriptome analysis in human islet grafts with induced islet cell death in a humanized mouse model, we identified the transcriptional landscape and regulation of lncRNAs associated with the deregulated mRNAs that are important for the molecular pathways and cellular processes related to the function and survival of human β cells and diabetes. We found 10 lnRNA isoforms that are highly enriched and specific to human islets. These islet-specific lncRNAs were characterized for the pattern of their subcellular localization, conservation status in mouse, and expression in the islets from the different categories of deceased human donors including T2D. Islet-specific lncRNAs were found to be deregulated in human islets in response to glucose-mediated induced insulin secretion and cytokine-mediated induced cell death, suggesting their potential role in human β-cell survival and function. To explore the possibility of using islet-specific lncRNAs as biomarkers of β-cell death and functional status, we identified the presence and regulation of islet-specific lncRNAs in the exosomes of islets secreted in the culture media *in vitro*. Collectively, we identified and provided evidence for the lncRNAs that might be important for human pancreatic islet cell death and survival and might have an impact on diabetes pathophysiology. Further functional analysis of those identified islet-specific lncRNAs, important for human β-cell function and survival, will help us identify therapeutic targets and biomarkers for β-cell loss.

## Data availability statement

The data presented in this study are publicly available at https://www.ncbi.nlm.nih.gov/sra/PRJNA1027842, under the BioProject/SRA accession number: PRJNA1027842.

## Ethics statement

The studies involving humans were approved by Institutional Animal Care and Use Committee. The studies were conducted in accordance with the local legislation and institutional requirements. Human islets were obtained through the Integrated Islet Distribution Program (IIDP, http://iidp.coh.org) sponsored by the National Institute of Diabetes and Kidney and Digestive Diseases (NIDDK). Written informed consent for participation was not required from the participants or the participants’ legal guardians/next of kin in accordance with the national legislation and institutional requirements. The animal study was approved by Institutional Animal Care and Use Committee at Sanford Research (protocol 77-08-16D). The study was conducted in accordance with the local legislation and institutional requirements.

## Author contributions

MH conducted the experiment, analyzed the data, and wrote the manuscript. RR, JC, CF, and FA assisted in conducting the experiments. BJ assisted in RNA sequencing and the initial bioinformatic analysis of the data. ZG designed the experiment, directed the study, and aided in writing the manuscript. All contributing authors have made necessary intellectual contributions to this work, and subsequently reviewed and approved it before submitting the final copy of the manuscript. All authors contributed to the article and approved the submitted version.

## Supplementary material

Data Sheet 1 Additional data.

Data Sheet 2 Additional results.
