# Supplementary material for: Exploring lncRNAs associated with human pancreatic islet cell death induced by transfer of adoptive lymphocytes in a humanized mouse model
Source: Front Endocrinol (Lausanne). 2023 Nov 1;14:1244688. doi: 10.3389/fendo.2023.1244688 (PMC10646418; doi:10.3389/fendo.2023.1244688)
Supplement: Data Sheet 1 — Additional data. [file DataSheet_1.pdf]

Additional data

Contents are arranged sequentially as it appeared in the manuscript entitled "Exploring lncRNAs Associated with Human Pancreatic Islet Cell Death Induced by Transfer of Adoptive Lymphocytes in a Humanized Mouse Model"

Table S1. List and level of enrichment of top canonical pathways identified to be affected by the differentially expressed mRNAs in ALT vs. PBS (CTL) using the IPA tool (Presented in Figure 2B).

Ingenuity Canonical Pathways	-log(p-value)	Ratio	Downregulated	No change	Upregulated	No overlap with dataset	Molecules
MIF Regulation of Innate Immunity	2.76E00	2.17E-01	6/23 (26%)	0/23 (0%)	11/23 (48%)	6/23 (26%)	TP53, RELA, MAPK1, NFKBIE, PLA2G10, PLA2G1B, MAPK8, NFKB1, TLR4, PLA2G6, FOS, LY96, PLA2G2F, MAPK3, PLA2G5, PTGS2, NOS2
Acute Phase Response Signaling	2.42E00	1.14E-01	11/79 (14%)	0/79 (0%)	44/79 (56%)	24/79 (30%)	RAF1, MAPK1, PIK3R1, SOCS6, HRAS, CP, IL6, MAPK13, IKBKB, ITIH2, ITIH4, MAPK3, MRAS, OSMR, IL1RAP, TAB1, AKT2, STAT3, TCF3, IL33, IL18, PTPN11, IL1RN, TNF, RBP4, MAP2K6, IL6ST, RELA, IL1A, TCF4, FN1, NFKBIE, NFKB1, MAPK11, NR3C1, HNRNPK, SHC1, HMOX1, MTOR, NGFR, SOS1, RBP2, SOCS2, AKT3, AGT, MYD88, TNFRSF1A, MAPK8, SERPINF1, IL1R1, CEBPB, FOS, RRAS2, MAPK14, IL1B
OX40 Signaling Pathway	2.42E00	2.35E-01	5/17 (29%)	0/17 (0%)	8/17 (47%)	4/17 (24%)	B2M, RELA, TRAF3, HLA-DOA, NFKBIE, MAPK8, NFKB1, BCL2, BCL2L1, CD3G, HLA-DMA, HLA-DMB, HLA-DRA
MIF-mediated Glucocorticoid Regulation	2.23E00	2.11E-01	5/19 (26%)	0/19 (0%)	9/19 (47%)	5/19 (26%)	PLA2G6, TLR4, RELA, LY96, PLA2G2F, MAPK1, MAPK3, NFKBIE, PLA2G10, PLA2G5, PLA2G1B, PTGS2, NFKB1, NR3C1
iNOS Signaling	2.23E00	1.67E-01	6/30 (20%)	0/30 (0%)	16/30 (53%)	8/30 (27%)	RELA, CAMK4, MAPK1, MYD88, NFKBIE, TYK2, HMGA1, MAPK13, NFKB1, MAPK11, IRF1, CALM1 (includes others), IKBKB, TLR4, FOS, LY96, MAPK14, STAT1, NOS2, JAK3, IRAK4, TAB1
JAK/Stat Signaling	1.58E00	1.16E-01	9/43 (21%)	0/43 (0%)	23/43 (53%)	11/43 (26%)	RELA, RAF1, PIAS2, MAPK1, PIK3R1, SOCS6, PIAS1, HRAS, IL6, NFKB1, SHC1, MTOR, MAPK3, CISH, SOS1, MRAS, SOCS2, AKT3, STAT1, CCKBR, AKT2, PTPN6, TYK2, STAT3, CEBPB, STAT4, FOS, BCL2L1, RRAS2, PTPN11, CDKN1A, JAK3
TNFR2 Signaling	1.56E00	1.76E-01	0/17 (0%)	0/17 (0%)	11/17 (65%)	6/17 (35%)	TANK, IKBKB, RELA, FOS, NFKBIE, MAPK8, TNFAIP3, NFKB1, TNF, BIRC2, TRAF1
Triacylglycerol Degradation	1.49E00	1.67E-01	8/18 (44%)	0/18 (0%)	2/18 (11%)	8/18 (44%)	FAAH, AADAC, ABHD6, CEL, PNLIPRP1, LIPE, MGLL, LIPG, NDST4, NDST1
Oncostatin M Signaling	1.43E00	1.58E-01	4/19 (21%)	0/19 (0%)	13/19 (68%)	2/19 (11%)	IL6ST, RAF1, EPAS1, MAPK1, TYK2, HRAS, STAT3, SHC1, RRAS2, MAPK3, SOS1, MRAS, OSMR, PLAU, STAT1, JAK3, MMP1
IL-10 Signaling	1.36E00	1.18E-01	2/34 (6%)	0/34 (0%)	25/34 (74%)	7/34 (21%)	MAP2K6, RELA, IL1A, MAPK1, IL1RL1, NFKBIE, IL6, MAPK13, NFKB1, MAPK11, IL1R2, IKBKB, HMOX1, IL1RAP, TAB1, TYK2, MAPK8, IL1R1, STAT3, IL33, FOS, IL18, MAPK14, IL1RN, IL1B, IL1RAPL1, TNF
Gluconeogenesis I	1.24E00	2E-01	2/10 (20%)	0/10 (0%)	6/10 (60%)	2/10 (20%)	GPI, ME3, ME2, FBP1, ALDOA, GAPDH, MDH2, ALDOC
Antigen Presentation Pathway	1.24E00	2E-01	5/10 (50%)	0/10 (0%)	4/10 (40%)	1/10 (10%)	B2M, CALR, HLA-DOA, PDIA3, HLA-DMA, HLA-DRA, HLA-DMB, CANX, TAPBP
Type I Diabetes Mellitus Signaling	1.13E00	8.77E-02	17/57 (30%)	0/57 (0%)	24/57 (42%)	16/57 (28%)	MAP2K6, RELA, HLA-DOA, MAPK1, GZMB, NFKBIE, SOCS6, PIAS1, MAPK13, NFKB1, MAPK11, BCL2, IKBKB, GAD2, CASP9, HLA-DMA, NGFR, HLA-DMB, HLA-DRA, SOCS2, NOS2, CASP8, STAT1, IL1RAP, PTPRN, FASLG, ICA1, CASP3, MYD88, TNFRSF1A, MAPK8, APAF1, IL1R1, HSPD1, IRF1, CD3G, MAPK14, GAD1, CD86, IL1B, TNF
Apoptosis Signaling	9.42E-01	8.51E-02	11/47 (23%)	0/47 (0%)	24/47 (51%)	12/47 (26%)	RAF1, RELA, BAD, MAPK1, NFKBIE, HRAS, NFKB1, BCL2, DIABLO, IKBKB, CASP9, MAPK3, MRAS, HTRA2, CASP8, FASLG, CAPN10, TP53, CASP3, TNFRSF1A, APAF1, MAPK8, BIRC6, BAX, PARP1, BCL2L1, CAPNS1, RRAS2, CASP2, DFFB, RPS6KA1, TNF, BCL2L11, CASP7, BIRC2

Table S2. Disease pathways regulated by the aberrantly expressed mRNAs between ALT-treated islet grafts and PBS (CTL)-treated islet grafts (Analysed by IPA tool and presented in Figure 2C).

Category	p-value	Molecules	Category	p-value	Molecules
Cell Death and Survival	1.49E-02-3.97E-02	IAPP, DIABLO, CEL, RELA, BCL2L1, CDH1, LEP, NDRG1	Endocrine System Disorders	3.97E-02-3.97E-02	UCHL1
Carbohydrate Metabolism	3.97E-02-3.97E-02	GCG	Energy Production	3.97E-02-3.97E-02	NAMPT
Cell Cycle	3.97E-02-3.97E-02	CEL	Gene Expression	3.97E-02-3.97E-02	RELA
Cell Signaling	3.97E-02-3.97E-02	GCG, AGT	Lipid Metabolism	3.97E-02-3.97E-02	GCG
Cellular Compromise	3.97E-02-3.97E-02	GCG	Metabolic Disease	3.97E-02-3.97E-02	UCHL1
Cellular Function and Maintenance	3.97E-02-3.97E-02	NDRG1	Molecular Transport	3.97E-02-3.97E-02	NAMPT, GCG, AGT

Table S3. MSigDB pathways enriched for the differentially expressed mRNAs from closely located mRNA-lncRNA pairs in ALT vs PBS (Presented in Figure 4D).

# Term Name	Binom Rank	Binom Raw PValue	Binom FDR QVal	Binom Fold Enrichment	Binom Observed Region Hits	Binom Region Set Coverage	Hyper Rank	Hyper FDR QVal	Hyper Fold Enrichment	Hyper Observed Gene Hits	Hyper Total Genes	Hyper Gene Set Coverage
Genes involved in Gene Expression	1	2.84706e16	2.50541e13	2.3409	114	7.16%	4	1.50420e4	1.6191	97	433	3.95%
Genes involved in Translation	2	1.90965e15	8.40246e13	4.4675	43	2.70%	9	1.63409e3	2.0567	35	123	1.43%
Genes involved in Diabetes pathways	3	7.40915e15	2.17335e12	2.2487	112	7.04%	1	4.36848e5	1.7400	91	378	3.71%
Genes involved in Influenza Viral RNA Transcription and rep	4	1.83424e14	4.03532e12	4.8187	37	2.32%	24	2.70013e2	1.8764	27	104	1.10%
Genes involved in GTP hydrolysis and joining of the 60S rs	5	3.82365e14	6.72962e12	4.4670	39	2.45%	12	3.67666e3	2.0556	31	109	1.26%
Genes involved in Influenza Life Cycle	6	6.88220e14	1.00939e11	3.9331	44	2.76%	26	2.75479e2	1.7306	34	142	1.38%
Genes involved in Metabolism of proteins	7	1.02300e13	1.28605e11	3.1402	58	3.64%	23	2.16840e2	1.5988	48	217	1.96%
Genes involved in Peptide chain elongation	8	4.25399e13	4.67939e11	4.9888	32	2.01%	16	1.19122e2	2.0769	25	87	1.02%
Genes involved in Glucose Regulation of Insulin Secretion	10	1.68875e12	1.48610e10	3.0316	55	3.45%	8	1.28178e3	1.9625	41	151	1.67%
Genes involved in Formation of a pool of free 40S subunits	11	3.34777e12	2.67821e10	4.3459	34	2.14%	17	1.37159e2	1.9913	27	98	1.10%
Oxidative phosphorylation	12	8.08169e12	5.92657e10	3.5877	41	2.58%	10	2.09661e3	2.0740	33	115	1.34%
Genes involved in Integration of energy metabolism	13	1.44108e11	9.75497e10	2.5440	67	4.21%	13	3.95843e3	1.7084	52	220	2.12%
Genes involved in Viral mRNA Translation	14	2.23034e11	1.40193e9	4.5358	30	1.88%	27	4.40901e2	1.9108	23	87	0.94%
Huntington's disease	15	2.48936e11	1.46043e9	2.7579	57	3.58%	6	3.35066e4	1.9750	47	172	1.91%
Genes involved in Regulation of Insulin Secretion	17	1.12688e10	5.83326e9	2.4018	68	4.27%	5	1.84010e4	1.9226	54	203	2.20%
Alzheimer's disease	19	3.63060e10	1.68154e8	2.6158	55	3.45%	2	1.22072e4	2.1312	46	156	1.87%
Circadian rhythm mammal	22	1.69197e9	6.76789e8	8.7872	14	0.88%	3	1.42100e4	5.5598	10	13	0.41%
Genes involved in Regulation of gene expression in beta cells	23	3.88054e9	1.48473e7	3.2258	35	2.20%	18	1.52953e2	1.9459	28	104	1.14%
Genes involved in Formation of ATP by chemiosmotic coupling	25	4.87074e9	1.71450e7	17.2387	9	0.57%	14	5.60541e3	4.4478	8	13	0.33%
Genes involved in Translation initiation complex formation	26	6.97088e9	2.35937e7	4.2608	24	1.51%	21	1.93479e2	2.2824	18	57	0.73%

The test set of 1,592 genomic regions picked 2,455 genes which are differentially expressed between ALT vs PBS (14%) of all 17,744 genes. MSigDB Pathway has 880 terms covering 6,673 (38%) of all 17,744 genes. 880 ontology terms were tested (100%) using an annotation count range of [1, Inf].

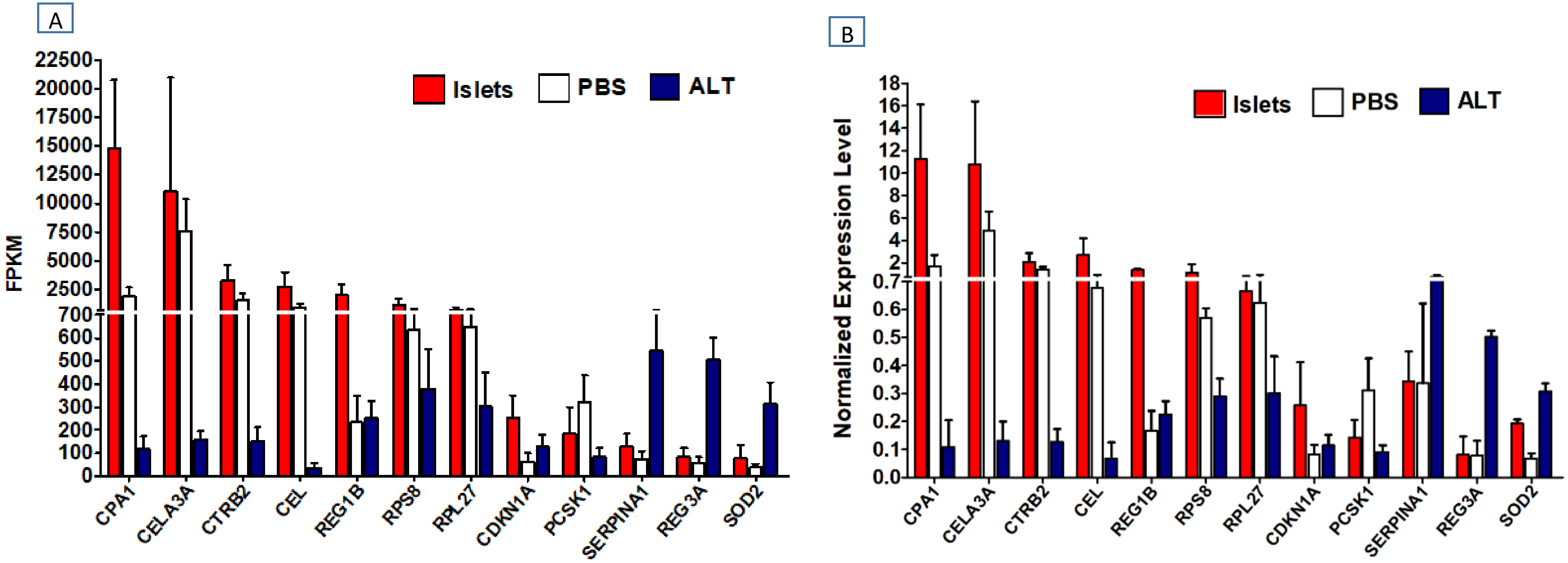

Figure S1: Validation of expression of 12 selected mRNAs in Seq analysis (A) using qRT-PCR (B).
The pattern and expression level of the top 12 islet-enriched mRNAs in islets, PBS-treated islet grafts and ALT-treated islet grafts. FPKM: Fragments per Kilobase per Million Mapped Reads. GAPDH and ACTB were used as endogenous controls for the measuring the relative expression of the same 12 mRNAs by qRT PCR. GAPDH and ACTB were used as endogenous controls for the measuring the relative expression of the same 10 lncRNAs by qRT PCR. Normalized expression level plotted on y-axis as 2^(-Average(Delta Ct) value for the lncRNAs in islets, PBS-treated islet grafts, and ALT-treated islet grafts. Normalization was performed against the mean expression value of human GAPDH and ACTB.

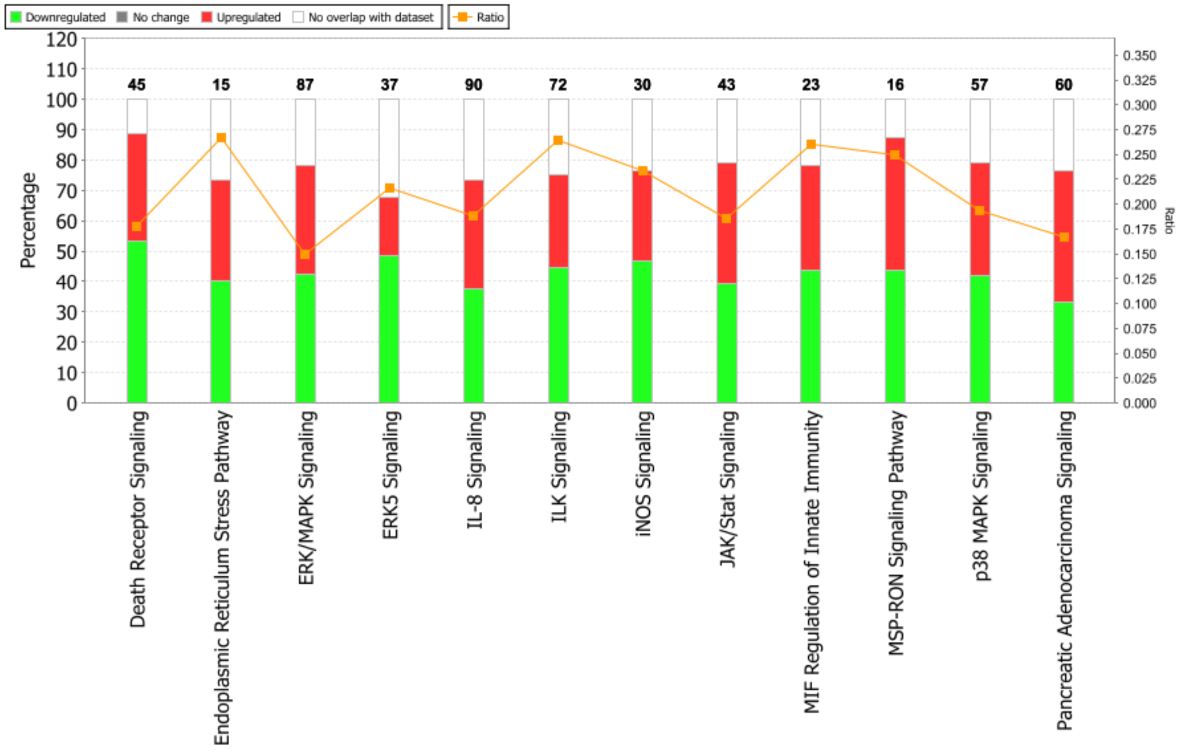

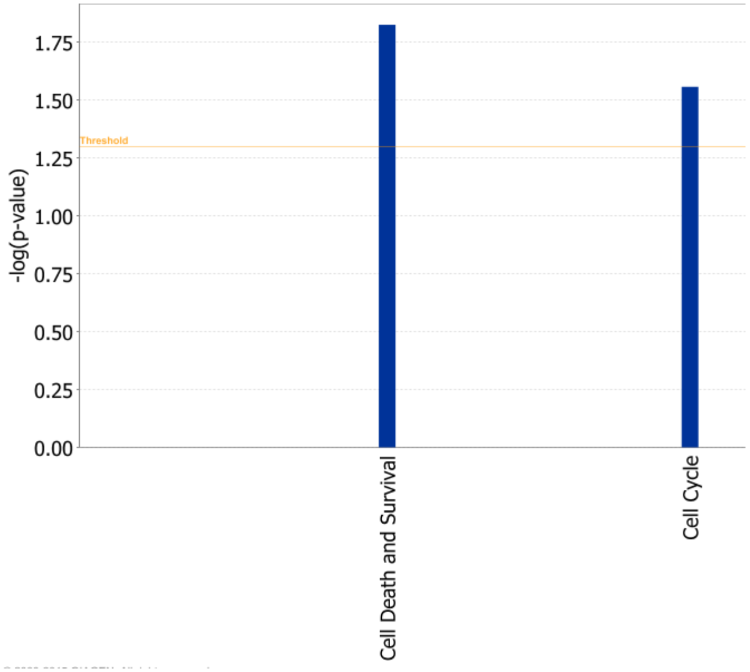

Figure S2: The most enriched molecular network of aberrantly expressed mRNA between PBS-treated islet grafts and islets.

Figure S3: The most affected disease and functional regulation by the aberrantly expressed mRNAs between PBS-treated islet grafts and islets.

List and level of enrichment of top molecular network and diseases-functional regulation identified to be affected by the differentially expressed mRNAs in PBS-treated islet grafts vs islets using the IPA tool. The figure displays the most significant network/disease-functional regulation across the differentially expressed mRNA. Ratio: percentage of transcripts in the pathway. Threshold: pathways with a $-\log_{10}(P) > 1.301$ were considered significantly regulated.

Table S4. The most enriched molecular network of aberrantly expressed mRNA between PBS-treated islet grafts and islets (Analysed by IPA tool and presented in Figure S2).

Ingenuity Canonical Pathways (PBS vs Islet)	-log(p-value)	Ratio	Downregulated	No change	Upregulated	No overlap with dataset	Molecules
Death Receptor Signaling	1.38E00	1.78E-01	24/45 (53%)	0/45 (0%)	16/45 (36%)	5/45 (11%)	HSPB3, RELA, HSPB2, NFKBIE, TNFSF10, NFKB1, FAS, BCL2, TANK, DIABLO, IKBKB, IKBKG, TNKS, CASP9, CRADD, TIPARP, TNFSF12, TNFSF15, HTRA2, CASP8, FASLG, MAP2K7, CASP3, TNFRSF1A, ACTB, PARP10, TNFRSF10B, MAPK8, APAF1, XIAP, PARP1, DAXX, ACTA2, CASP2, DFFB, CFLAR, TNF, CASP7, BIRC2, HSPB1
Endoplasmic Reticulum Stress Pathway	1.42E00	2.67E-01	6/15 (40%)	0/15 (0%)	5/15 (33%)	4/15 (27%)	CALR, CASP9, CASP3, DDIT3, ERN1, MAPK8, XBP1, ATF4, EIF2AK3, CASP7, TAOK3
ERK/MAPK Signaling	1.38E00	1.49E-01	37/87 (43%)	0/87 (0%)	31/87 (36%)	19/87 (22%)	PRKACB, RAPGEF1, RAF1, RAC2, YWHAH, MAPK1, BAD, PPP2CA, HSPB2, PIK3R1, HRAS, MYC, ITGA3, PAK1, ELF3, ETS2, PIK3CG, MAPK3, MRAS, ATF4, ETS1, RAC1, YWHAZ, MAPKAPK5, RAPGEF3, STAT3, RAP1A, RAC3, ELF1, DOCK1, PPP2CB, PLA2G6, PPP2R1A, H3F3A/H3F3B, PRKCD, PRKACA, RPS6KA1, ESR1, HSPB1, HSPB3, PPP2R2A, PLA2G10, RAPGEF4, BCAR1, DUSP2, NFATC1, EIF4EBP1, BRAF, SHC1, ELF4, PLA2G2F, SOS1, MKNK1, STAT1, PRKCA, PPARG, ITGB1, SRC, PAK4, PAK6, PLA2G1B, RPS6KA5, PLA2G4A, FOS, RRAS2, PRKCI, PRKAR1A, PRKCB
ERK5 Signaling	1.86E00	2.16E-01	18/37 (49%)	0/37 (0%)	7/37 (19%)	12/37 (32%)	IL6ST, RPS6KB1, SRC, BAD, YWHAH, SGK1, YWHAZ, HRAS, RPS6KA5, MYC, FOS, RRAS2, AKT1, GAB1, PTPN11, NTRK1, RPS6KB2, MRAS, ATF4, FOSL1, RPS6KA1, GNA13, RPS6KA2, WNK1, EGFR
IL-8 Signaling	2.7E00	1.89E-01	34/90 (38%)	0/90 (0%)	32/90 (36%)	24/90 (27%)	RAF1, RAC2, PLD2, MAPK1, DIRAS3, PIK3R1, GNB2L1, GNB5, HRAS, CCND1, VEGFA, IKBKB, IKBKG, CCND3, RHOB, PIK3CG, MAPK3, MRAS, FIGF, GNA13, PRKD3, PRKD1, CR2, RPS6KB1, NOX4, AKT2, GNG2, RAC1, HBEGF, MMP2, RAC3, PLD1, BCL2L1, CDH1, RND3, PRKCD, RELA, NOX3, RHOT2, VEGFB, NFKB1, EIF4EBP1, BCL2, BRAF, HMOX1, JUN, AKT1, AKT3, GNB1L, LASP1, PRKCA, EGFR, SRC, GNAS, FLT1, RHOC, MAPK8, BAX, GNAI2, FOS, RRAS2, PRKCI, PTGS2, KDR, PRKCB, IRAK2
ILK Signaling	5.01E00	2.64E-01	32/72 (44%)	0/72 (0%)	22/72 (31%)	18/72 (25%)	MAPK1, PPP2CA, DIRAS3, PIK3R1, MUC1, PDPK1, CCND1, MYC, VEGFA, RHOB, PIK3CG, MAPK3, FIGF, ATF4, MYL4, IRS2, TESK1, DSP, AKT2, CASP3, MYH14, PPP2CB, DOCK1, CDH1, PPP2R1A, ACTA2, RND3, KRT18, TNF, MAP2K6, FLNB, RELA, FN1, PPP2R2A, RHOT2, VEGFB, HIF1A, NFKB1, JUN, AKT1, FLNA, AKT3, NOS2, CTNNB1, NACA, ITGB1, FBLIM1, TNFRSF1A, RHOC, ACTB, MAPK8, RPS6KA5, FOS, PTGS2
iNOS Signaling	1.86E00	2.33E-01	14/30 (47%)	0/30 (0%)	9/30 (30%)	7/30 (23%)	RELA, CAMK4, MAPK1, MYD88, NFKBIE, TYK2, HMGA1, MAPK13, NFKB1, MAPK11, IRF1, CALM1 (includes others), IKBKB, TLR4, FOS, LY96, IKBKG, MAPK14, JUN, STAT1, NOS2, JAK3, IRAK2
JAK/Stat Signaling	1.49E00	1.86E-01	17/43 (40%)	0/43 (0%)	17/43 (40%)	9/43 (21%)	SOCS3, RAF1, RELA, MAPK1, PIK3R1, PIAS1, HRAS, NFKB1, SHC1, AKT1, JUN, PIK3CG, MAPK3, CISH, SOS1, MRAS, SOCS2, AKT3, STAT1, CCKBR, AKT2, PTPN6, TYK2, SOCS4, STAT3, CEBPB, STAT4, BCL2L1, FOS, GAST, RRAS2, PTPN11, CDKN1A, JAK3
MIF Regulation of Innate Immunity	1.89E00	2.61E-01	10/23 (43%)	0/23 (0%)	8/23 (35%)	5/23 (22%)	TP53, RELA, MAPK1, NFKBIE, PLA2G10, PLA2G1B, MAPK8, NFKB1, FOS, TLR4, PLA2G6, PLA2G4A, LY96, JUN, PLA2G2F, MAPK3, PTGS2, NOS2
MSP-RON Signaling Pathway	1.33E00	2.5E-01	7/16 (44%)	0/16 (0%)	7/16 (44%)	2/16 (13%)	KLKB1, TLR4, CSF2RB, ACTA2, KLK3, CSF1, PIK3CG, ACTB, PIK3R1, KLK1, MST1R, RPS6KA2, NOS2, TNF
p38 MAPK Signaling	1.99E00	1.93E-01	24/57 (42%)	0/57 (0%)	21/57 (37%)	12/57 (21%)	HSPB3, MAP2K6, IL1A, TGFBR1, DDIT3, IL1RL1, HSPB2, PLA2G10, HMGN1, MAPK13, MAPK11, FAS, MYC, CDC25B, IL1R2, PLA2G2F, MKNK1, RPS6KB2, ATF4, RPS6KA2, STAT1, IL1RAP, FASLG, TP53, RPS6KB1, MAPKAPK3, TNFRSF1A, PLA2G1B, MAPKAPK5, RPS6KA5, IL1R1, IL33, PLA2G4A, PLA2G6, DAXX, IL18, MAPK14, H3F3A/H3F3B, IL1RN, IL1B, IL1RAPL1, RPS6KA1, TNF, HSPB1, IRAK2
Pancreatic Adenocarcinoma Signaling	1.44E00	1.67E-01	20/60 (33%)	0/60 (0%)	26/60 (43%)	14/60 (23%)	CDKN2A, RAF1, RELA, PLD2, TGFBR1, BAD, MAPK1, PIK3R1, SMAD3, CDK4, VEGFB, NFKB1, CCND1, BCL2, VEGFA, E2F6, RB1, HMOX1, CASP9, AKT1, MAPK3, PIK3CG, SMAD4, FIGF, AKT3, ERBB2, STAT1, EGFR, TP53, SMAD2, AKT2, TFDP1, TYK2, RAC1, MAPK8, HBEGF, MDM2, STAT3, BIRC5, PLD1, BCL2L1, CDKN1A, TGFA, PTGS2, JAK3, CDK2

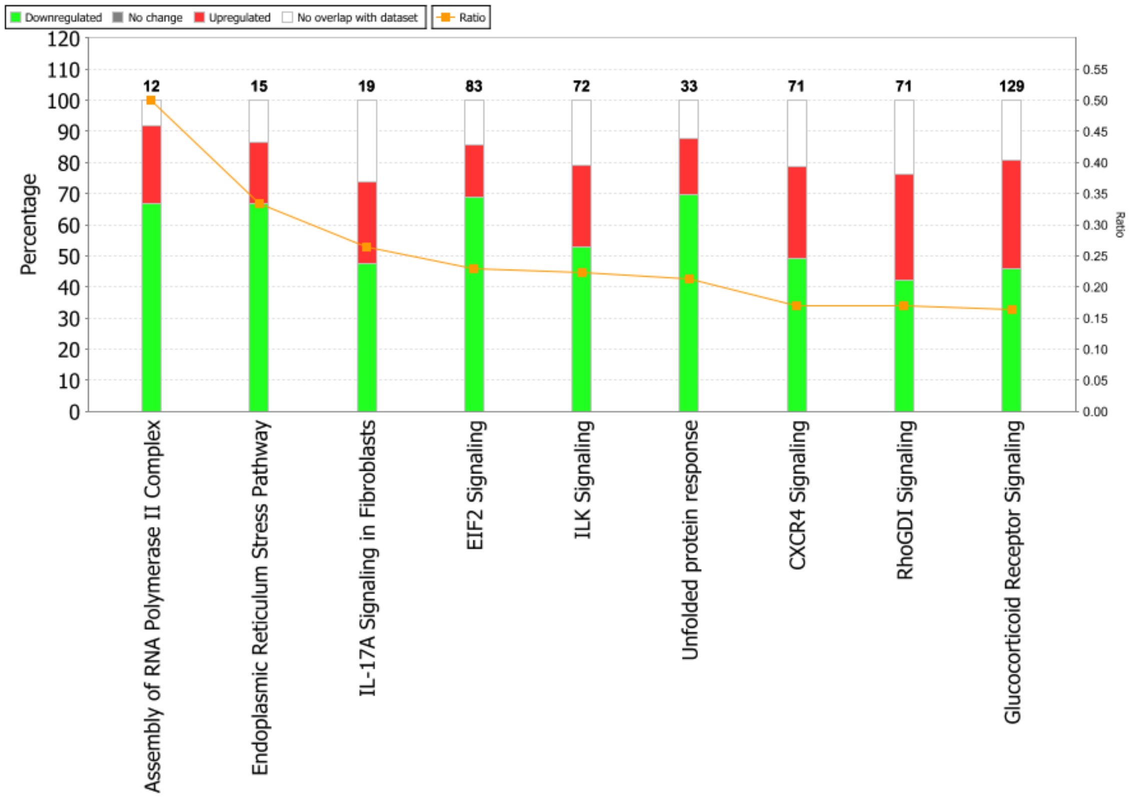

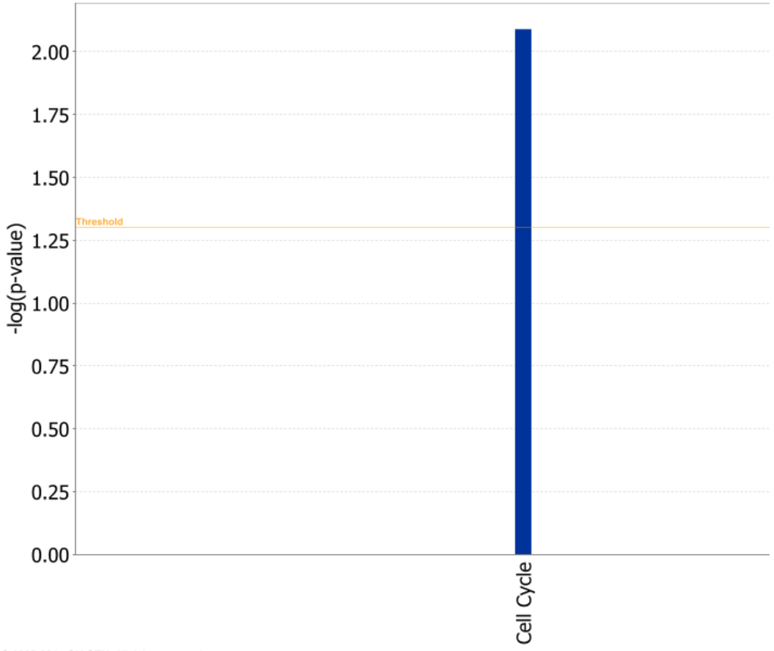

Figure S4: The most enriched molecular network of aberrantly expressed mRNA between ALT-treated islet grafts and islets.

Figure S5: The most affected disease and functional regulation by the aberrantly expressed mRNAs between ALT-treated islet grafts and islets.

List and level of enrichment of top molecular network and diseases-functional regulation identified to be affected by the differentially expressed mRNAs in ALT-treated islet grafts vs islets using the IPA tool. The figure displays the most significant network/disease-functional regulation across the differentially expressed mRNA. Ratio: percentage of transcripts in the pathway. Threshold: pathways with a $-\log_{10}(P) > 1.301$ were considered significantly regulated.

Table S5. The most enriched molecular network of aberrantly expressed mRNA between ALT-treated islet grafts and islets (Analysed by IPA tool and presented in Figure S4).

Ingenuity Canonical Pathways (PBS vs Islet)	-log(p-value)	Ratio	Downregulated	No change	Upregulated	No overlap with dataset	Molecules
Assembly of RNA Polymerase II Complex	3.29E00	5E-01	8/12 (67%)	0/12 (0%)	3/12 (25%)	1/12 (8%)	POLR2G, GTF2A2, TAF12, TAF9, GTF2B, POLR2D, TAF6, POLR2C, POLR2J, GTF2H1, GTF2A1
Endoplasmic Reticulum Stress Pathway	1.91E00	3.33E-01	10/15 (67%)	0/15 (0%)	3/15 (20%)	2/15 (13%)	CALR, CASP3, DDIT3, ERN1, MAPK8, XBP1, HSPA5, TAOK3, HSP90B1, CASP9, ATF4, EIF2AK3, CASP7
IL-17A Signaling in Fibroblasts	1.46E00	2.63E-01	9/19 (47%)	0/19 (0%)	5/19 (26%)	5/19 (26%)	RELA, MAPK1, NFKBIE, IL6, NFKB1, MAPK11, NFKBID, IKBKB, FOS, JUN, MAPK14, MAPK3, NFKBIB, MMP1
EIF2 Signaling	3.42E00	2.29E-01	57/83 (69%)	0/83 (0%)	14/83 (17%)	12/83 (14%)	RAF1, EIF3C, MAPK1, PIK3R1, PDPK1, HRAS, EIF4A2, EIF2A, RPS7, EIF1, EIF3B, EIF3D, MAPK3, PIK3CG, EIF5, RPL19, MRAS, RPL21, PPP1CA, PABPC1, EIF2AK1, AKT2, RPL36AL, RPL3, RPL12, EIF3E, RPL37A, RPL28, RPL10A, RPL15, RPL8, EIF3K, EIF2B4, RPS18, RPL26, EIF4G1, SHC1, RPL35, AKT1, SOS1, EIF3A, AKT3, RPS17, RPS3, RPS5, RPL18, RPL31, RPS24, RPS19, RPL4, EIF3H, RPL30, AGO2, EIF3J, RPS21, FAU, EIF3G, RPL27, EIF3F, RPS15, RPS16, RRAS2, RPS27L, EIF3I, RPS27A, EIF2AK2, EIF2AK3, EIF3L, RPS14, RPL38, RPLP0
ILK Signaling	2.82E00	2.22E-01	38/72 (53%)	0/72 (0%)	19/72 (26%)	15/72 (21%)	MAPK1, PIK3R1, MUC1, PDPK1, PPP1R14B, CCND1, MYC, NCK2, VEGFA, RHOB, PIK3CG, MAPK3, FIGF, ATF4, IRS2, TESK1, AKT2, CASP3, MYH14, SNAI1, PPP2CB, DOCK1, PPP2R1A, CDH1, RHOQ, ACTA2, RND3, CDC42, MAP2K6, FLNB, RELA, FN1, SNAI2, PPP2R2A, RHOT2, VEGFB, HIF1A, NFKB1, MTOR, JUN, AKT1, FLNA, AKT3, CTNNB1, NACA, ITGB1, FBLIM1, TNFRSF1A, RHOC, ACTB, MAPK8, VIM, RPS6KA5, PPP2R5A, FOS, PTGS2, PPP2R5E
Unfolded protein response	1.39E00	2.12E-01	23/33 (70%)	0/33 (0%)	6/33 (18%)	4/33 (12%)	DDIT3, INSIG1, ERN1, OS9, HSPA5, EIF2A, BCL2, SYVN1, HSP90B1, UBXN4, VCP, ATF4, DNAJA2, NFE2L2, PPARG, CALR, PDIA2, P4HB, MAP2K7, HSPA9, XBP1, MAPK8, CANX, SEL1L, HSPA8, SREBF1, SREBF2, ERO1LB, EIF2AK3
CXCR4 Signaling	1.34E00	1.69E-01	35/71 (49%)	0/71 (0%)	21/71 (30%)	15/71 (21%)	RAF1, MAPK1, CD4, PIK3R1, GNB2L1, CXCL12, GNB5, HRAS, GNB1, PAK1, RHOB, MAPK3, PIK3CG, MRAS, PLCB1, ARHGEF11, GNA13, PRKD3, PRKD1, AKT2, GNG2, ITPR1, ADCY9, DOCK1, RHOQ, RND3, PRKCD, ITPR3, GNAT2, PAK7, ELMO2, ELMO1, GNA11, RHOT2, CRK, GNA14, BCAR1, JUN, AKT1, AKT3, GNB1L, PRKCA, SRC, PAK4, GNAS, PAK6, RHOC, CXCR4, EGR1, MAPK8, GNAI2, FOS, RRAS2, ADCY1, ADCY7, PRKCB
RhoGDI Signaling	1.34E00	1.69E-01	30/71 (42%)	0/71 (0%)	24/71 (34%)	17/71 (24%)	GNB2L1, GNB5, GNB1, ITGA3, PAK1, RHOB, MRAS, ARHGEF11, GNA13, ACTR2, ARHGEF4, ARHGEF15, GNG2, ARHGEF17, WASF1, ARHGAP5, CDH2, CDH1, RHOQ, ACTA2, RND3, CDC42, ARPC2, CDH20, GNAT2, PAK7, ARHGEF18, ESR1, GDI1, PPP1R12C, RHOT2, GNA11, GNA14, CDH7, ACTR3, ARPC3, GNB1L, PI4KA, PRKCA, ITGB1, SRC, PAK4, GNAS, CDH4, PAK6, RHOC, ACTB, GDI2, CDH15, GNAI2, ARHGEF5, CDH10, CD44, ARHGDIA
Glucocorticoid Receptor Signaling	1.81E00	1.63E-01	59/129 (46%)	0/129 (0%)	45/129 (35%)	25/129 (19%)	RAF1, TGFBR1, PRKAB1, SGK1, PIK3R1, HRAS, SMARCD2, ARID2, IL6, SUMO1, PIK3CG, SMARCB1, NFKBIB, SMAD2, AKT2, CDKN1C, MED1, STAT3, HSPA8, POU2F1, PRKACA, GTF2H1, POLR2J, SMARCA4, GTF2A2, AR, AKT1, JUN, NCOA2, SOS1, NCOR1, FKBP5, MMP1, PPP3CA, SRA1, ACTB, MAPK8, TSG101, FOS, VIPR1, CDKN1A, CSN2, NRIP1, PTGS2, PLAU, JAK3, PRKACB, POLR2D, MAPK1, YWHAH, NFATC3, SMAD3, PBX1, CD163, HSPA5, FCGR1A, TGFBR2, IKBKB, MAPK3, MRAS, TAB1, TAF9, HSPA9, SMARCD3, MED14, CD3G, BCL2L1, IL1RN, SMARCA2, NCOA1, HSP90AA1, ESR1, UBE2I, RELA, ARID1A, GTF2F2, NFKBIE, GTF2A1, NFKB1, MAPK11, NR3C1, NFATC1, BCL2, PPP3R2, SHC1, GTF2B, HSP90B1, NFAT5, POLR2C, ANXA1, FOXO3, SMAD4, AKT3, STAT1, TAF12, MAP2K7, TAF6, SMARCE1, POLR2G, RRAS2, MAPK14, IL1B, NFATC2, SMARCC1

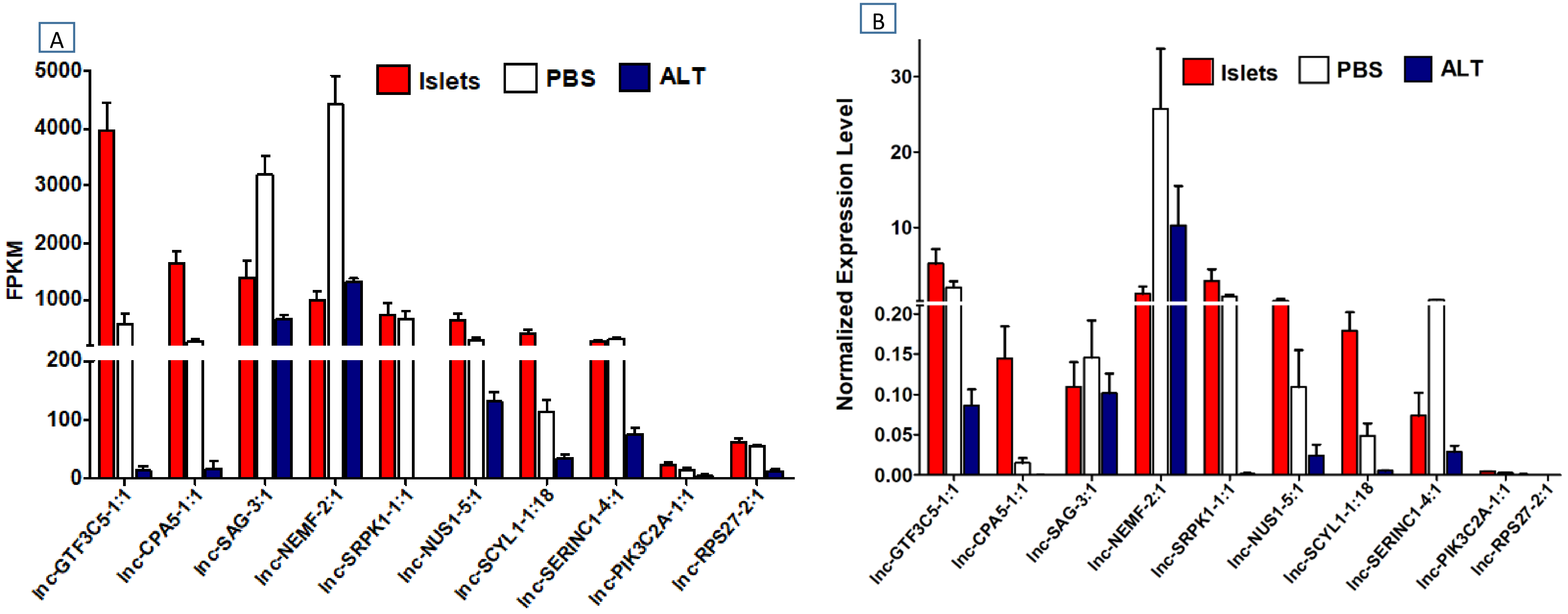

Figure S6: Expression levels of 10 lncRNAs identified by the sequencing (A) and validated by real time qRT-PCR (B).
The pattern and expression level of selected 10 highly islet-enriched lncRNAs in islets, PBS-treated islet grafts and ALT-treated islet grafts. FPKM: Fragments per Kilobase per Million Mapped Reads. GAPDH and ACTB were used as endogenous controls for the measuring the relative expression of the same 10 lncRNAs by qRT PCR. Normalized expression level plotted on y-axis as 2^(-Average(Delta Ct) value for the lncRNAs in islets, PBS-treated islet grafts, and ALT-treated islet grafts. Normalization was performed against the mean expression value of human GAPDH and ACTB.

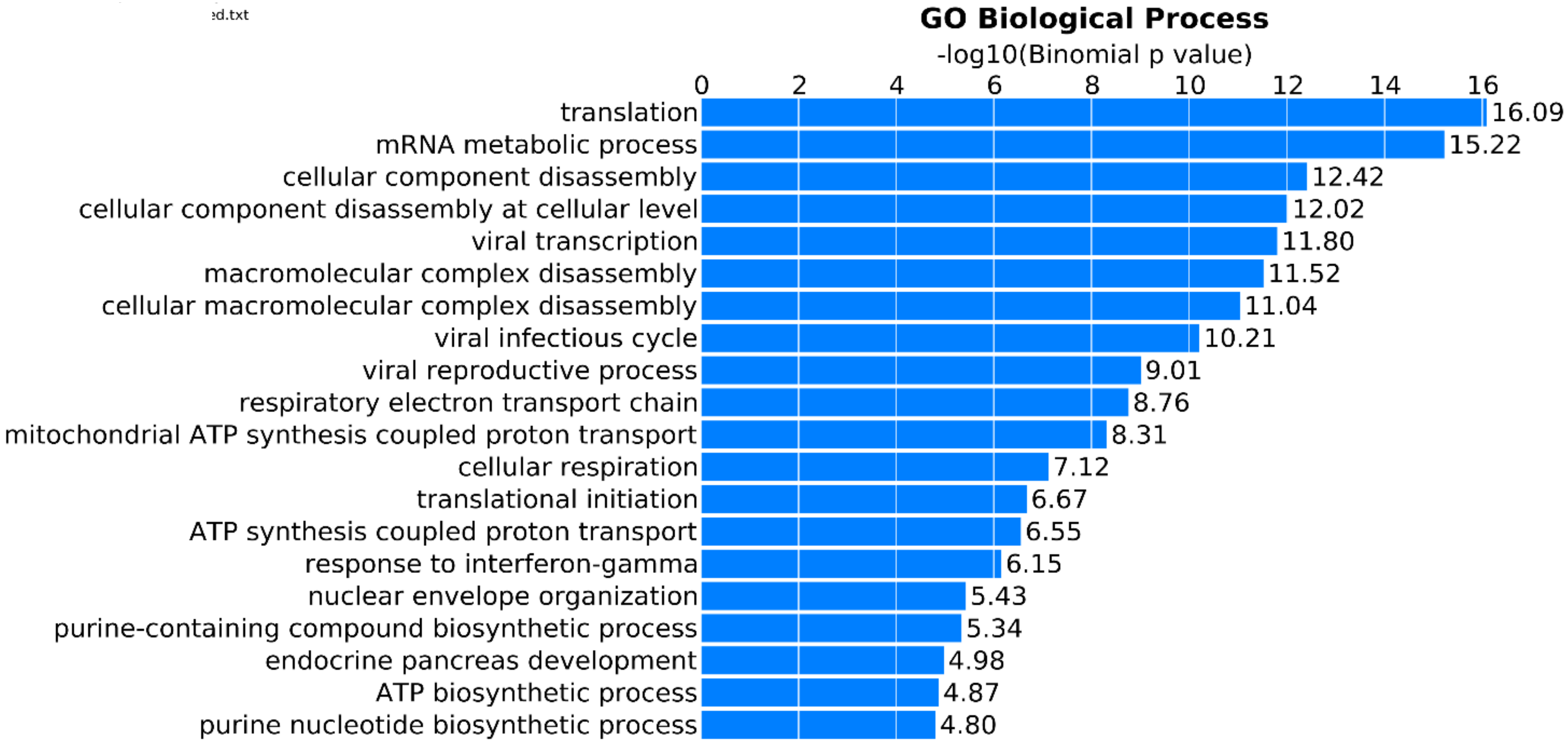

Figure S7: Highly enriched GO biological processes affected by 1145 matched mRNA-lncRNA pairs.
Genomic Regions Enrichment of Annotations Tool (GREAT) enrichment analysis of differentially regulated 1145 matched mRNA-lncRNA pairs based on biological process Gene Ontology (GO) terms. Enrichment analysis was performed using the GREAT algorithm (http://great. stanford.edu). The GREAT algorithm associates each human gene (n 17,744) with a regulatory domain in the human genome (hg19 assembly) and calculates the total fraction of the genome annotated with GO terms. The submitted sequences that fall in each annotated GO term region are counted as "hits." A binomial test compares the expected number of hits in a genome region with the observed number of hits. Listed in the figure are the most significantly enriched biological process GO terms (out of 8,761). Expected and observed counts for each GO term are listed with binomial test P value. Figure presents the top biological process GO terms with the highest p-value. (Details in Table S6).

Table S6. Highly enriched GO biological processes affected by 1145 matched mRNA-lncRNA pairs (Analysed by GREAT and presented in Figure S7).

# Term Name	Binom Rank	Binom Raw P Value	Binom FDR QVal	Binom Fold Enrichment	Binom Observed Region Hits	Binom Region Set Coverage	Hyper Rank	Hyper FDR QVal	Hyper Fold Enrichment	Hyper Observed Gene Hits	Hyper Total Genes	Hyper Gene Set Coverage
translation	9	8.06575e17	7.85156e14	2.6733	93	5.84%	56	4.83773e3	1.5677	77	355	3.14%
mRNA metabolic process	11	5.96570e16	4.75141e13	2.0606	146	9.17%	129	2.02280e2	1.3619	114	605	4.64%
cellular component disassembly	17	3.83998e13	1.97895e10	2.7429	68	4.27%	17	3.20111e4	1.9391	55	205	2.24%
cellular component disassembly at cellular level	20	9.58194e13	4.19737e10	2.7107	67	4.21%	21	5.14493e4	1.9132	54	204	2.20%
viral transcription	21	1.57382e12	6.56584e10	4.8956	31	1.95%	158	3.08282e2	1.9938	24	87	0.98%
macromolecular complex disassembly	22	3.00873e12	1.19816e9	3.5678	43	2.70%	47	3.76999e3	2.0401	35	124	1.43%
cellular macromolecular complex disassembly	25	9.14082e12	3.20331e9	3.5064	42	2.64%	64	5.78742e3	1.9979	34	123	1.38%
viral infectious cycle	32	6.21932e11	1.70273e8	3.1828	44	2.76%	175	3.56431e2	1.7428	34	141	1.38%
viral reproductive process	35	9.76122e10	2.44337e7	2.6852	50	3.14%	207	4.61110e2	1.6388	39	172	1.59%
respiratory electron transport chain	37	1.74244e9	4.12583e7	3.1999	37	2.32%	67	6.27009e3	2.1172	29	99	1.18%
mitochondrial ATP synthesis coupled proton transport	41	4.87074e9	1.04079e6	17.2387	9	0.57%	85	9.19152e3	4.4478	8	13	0.33%
cellular respiration	55	7.55223e8	1.20300e5	2.4943	44	2.76%	73	7.03379e3	1.9274	36	135	1.47%
translational initiation	63	2.12572e7	2.95610e5	3.4220	25	1.57%	198	4.30800e2	2.0238	21	75	0.86%
ATP synthesis coupled proton transport	66	2.79054e7	3.70423e5	9.0131	10	0.63%	123	1.94158e2	3.6138	9	18	0.37%
response to interferongamma	79	7.03358e7	7.80015e5	2.6751	33	2.07%	194	4.25776e2	1.9274	24	90	0.98%
nuclear envelope organization	93	3.72288e6	3.50711e4	9.1940	8	0.50%	110	1.51588e2	4.5994	7	11	0.29%
purinecontaining compound biosynthetic process	97	4.58766e6	4.14356e4	2.2487	39	2.45%	105	1.37242e2	1.8759	34	131	1.38%
endocrine pancreas development	115	1.05663e5	8.04972e4	2.1187	41	2.58%	117	1.74508e2	1.8634	33	128	1.34%
ATP biosynthetic process	124	1.34232e5	9.48390e4	3.3482	18	1.13%	109	1.50025e2	2.5076	17	49	0.69%
purine nucleotide biosynthetic process	129	1.56769e5	1.06470e3	2.2345	35	2.20%	126	2.02601e2	1.8828	31	119	1.26%

The test set of 1,592 genomic regions picked 2,455 genes which are differentially expressed between ALT vs PBS (14%) of all 17,744 genes. GO Biological Process has 8,761 terms covering 14,760 (83%) of all 17,744 genes. 8,761 ontology terms were tested (100%) using an annotation count range of [1, Inf].

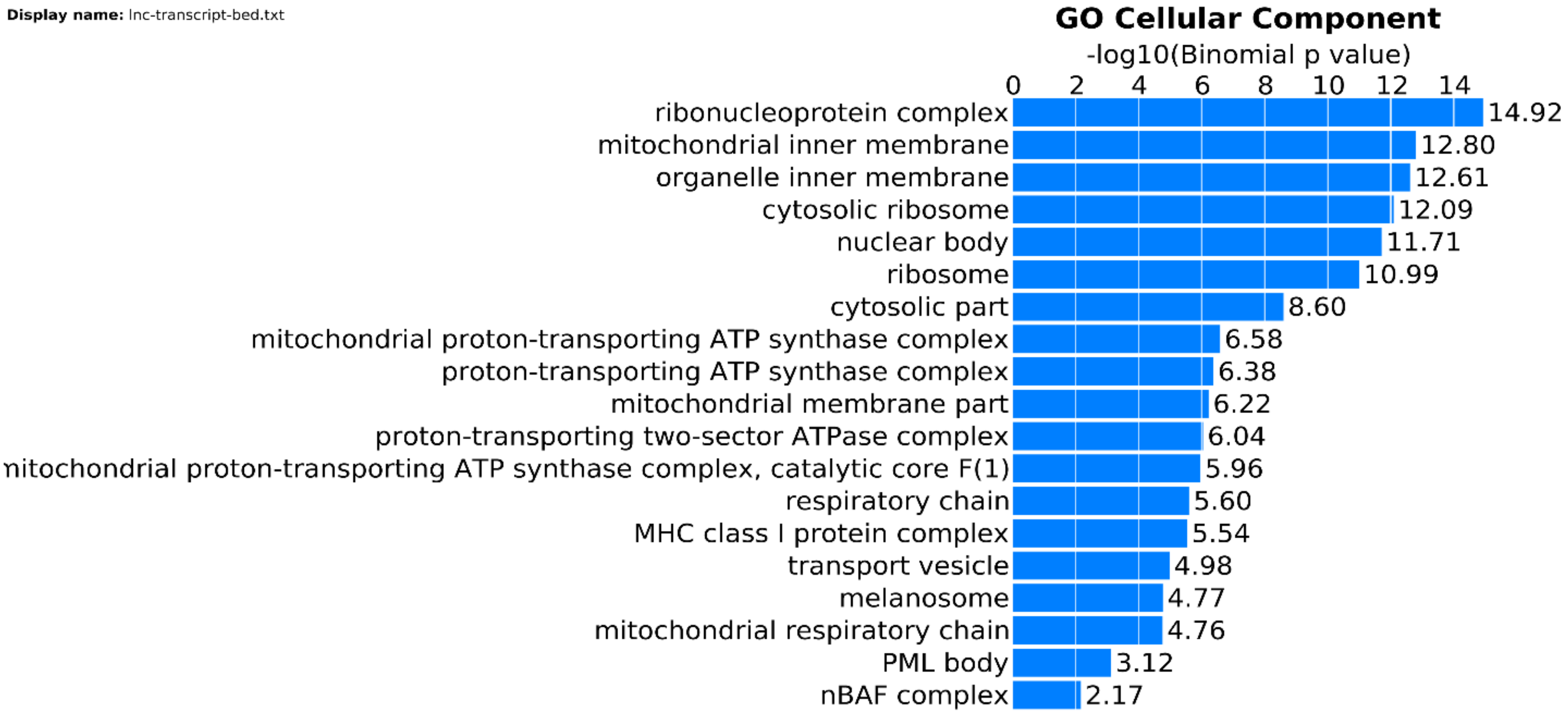

Figure S8: Highly enriched GO cellular components affected by the mRNA-lncRNA pairs in CIS regulation.

Genomic Regions Enrichment of Annotations Tool (GREAT) enrichment analysis of differentially regulated 1145 matched mRNA-lncRNA pairs based on biological process Gene Ontology (GO) terms. Enrichment analysis was performed using the GREAT algorithm (http://great. stanford.edu). The GREAT algorithm associates each human gene (n 17,744) with a regulatory domain in the human genome (hg19 assembly) and calculates the total fraction of the genome annotated with GO terms. The submitted sequences that fall in each annotated GO term region are counted as "hits." A binomial test compares the expected number of hits in a genome region with the observed number of hits. Listed in the figure are the most significantly enriched cellular components GO terms (out of 1,125). Expected and observed counts for each GO term are listed with binomial test P value. Figure presents the top cellular components GO terms with the highest p-value. (Details in Table S7).

Table S7. Highly enriched GO cellular components affected by the mRNA-lncRNA pairs in CIS regulation (Analysed by GREAT and presented in Figure S8).

# Term Name	Binom Rank	Binom Raw PValue	Binom FDR QVal	Binom Fold Enrichment	Binom Observed Region Hits	Binom Region Set Coverage	Hyper Rank	Hyper FDR QVal	Hyper Fold Enrichment	Hyper Observed Gene Hits	Hyper Total Genes	Hyper Gene Set Coverage
ribonucleoprotein complex	23	1.19242e15	5.83247e14	2.1670	128	8.04%	49	1.56048e2	1.3488	106	568	4.32%
mitochondrial inner membrane	25	1.60177e13	7.20794e12	2.4419	86	5.40%	34	5.17593e3	1.5285	70	331	2.85%
organelle inner membrane	26	2.47204e13	1.06963e11	2.3515	91	5.72%	33	4.75190e3	1.5100	75	359	3.05%
cytosolic ribosome	27	8.14658e13	3.39441e11	4.4560	35	2.20%	41	7.27777e3	2.0206	26	93	1.06%
nuclear body	28	1.95938e12	7.87250e11	2.4311	79	4.96%	27	6.99793e4	1.7073	60	254	2.44%
ribosome	30	1.01374e11	3.80152e10	2.7648	59	3.71%	62	4.04953e2	1.5181	46	219	1.87%
cytosolic part	37	2.53106e9	7.69578e8	2.5494	52	3.27%	39	7.12807e3	1.7076	43	182	1.75%
mitochondrial protontransporting ATP synthase complex	47	2.60751e7	6.24139e6	9.0812	10	0.63%	42	7.30146e3	3.6138	9	18	0.37%
protontransporting ATP synthase complex	48	4.20578e7	9.85729e6	8.6107	10	0.63%	46	1.11155e2	3.4236	9	19	0.37%
mitochondrial membrane part	50	6.04695e7	1.36056e5	2.6940	33	2.07%	61	3.68903e2	1.7073	30	127	1.22%
protontransporting twosector ATPase complex	51	9.08042e7	2.00303e5	4.5589	16	1.01%	67	4.75264e2	2.1997	14	46	0.57%
mitochondrial protontransporting ATP synthase complex	52	1.10295e6	2.38618e5	54.9105	4	0.25%	59	3.10089e2	5.7822	4	5	0.16%
respiratory chain	57	2.51451e6	4.96284e5	3.3621	21	1.32%	56	2.83697e2	2.0496	19	67	0.77%
MHC class I protein complex	58	2.88408e6	5.59412e5	7.9902	9	0.57%	64	4.20160e2	2.8282	9	23	0.37%
transport vesicle	64	1.03828e5	1.82510e4	2.1679	39	2.45%	40	6.96959e3	1.8988	31	118	1.26%
melanosome	67	1.71062e5	2.87232e4	2.3599	31	1.95%	47	1.57094e2	1.9429	25	93	1.02%
mitochondrial respiratory chain	68	1.74465e5	2.88637e4	3.1567	19	1.19%	70	4.84563e2	2.0143	17	61	0.69%
PML body	99	7.51465e4	8.53937e3	2.2799	20	1.26%	54	1.97258e2	2.1127	19	65	0.77%
nBAF complex	154	6.76902e3	4.94490e2	3.2385	7	0.44%	71	4.82572e2	3.6138	6	12	0.24%

The test set of 1,592 genomic regions picked 2,455 genes which are differentially expressed between ALT vs PBS (14%) of all 17,744 genes. GO Cellular Component has 1,125 terms covering 16,450 (93%) of all 17,744 genes. 1,125 ontology terms were tested (100%) using an annotation count range of [1, Inf].

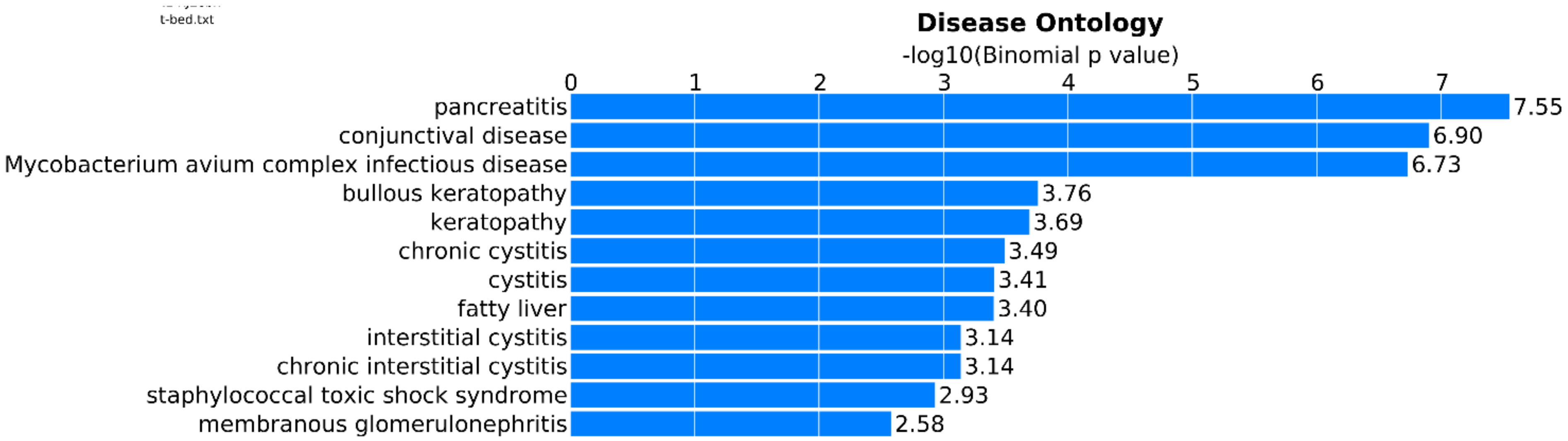

Figure S9: Disease ontology highly enriched for the deregulated mRNAs from the mRNA-lncRNAs pairs. Genomic Regions Enrichment of Annotations Tool (GREAT) enrichment analysis of differentially regulated 1145 matched mRNA-lncRNA pairs based on disease ontology GO terms. Enrichment analysis was performed using the GREAT algorithm (http://great. stanford.edu). The GREAT algorithm associates each human gene (n 17,744) with a regulatory domain in the human genome (hg19 assembly) and calculates the total fraction of the genome annotated with GO terms. The submitted sequences that fall in each annotated GO term region are counted as "hits." A binomial test compares the expected number of hits in a genome region with the observed number of hits. Listed in the figure are the most significantly enriched biological process GO terms (out of 2,233). Expected and observed counts for each GO term are listed with binomial test P value. Figure presents the top disease ontology GO terms with the highest p-value. (Details in Table S8).

Table S8. Disease ontology highly enriched for the deregulated mRNAs from the mRNA-lncRNAs pairs (Analysed by GREAT and presented in Figure S9).

# Term Name	Binom Rank	Binom Raw P Value	Binom FDR QVal	Binom Fold Enrichment	Binom Observed Region Hits	Binom Region Set Coverage	Hyper Rank	Hyper FDR QVal	Hyper Fold Enrichment	Hyper Observed Gene Hits	Hyper Total Genes	Hyper Gene Set Coverage
pancreatitis	13	2.82750e8	4.85677e6	2.7751	39	2.45%	142	4.06228e2	1.7150	28	118	1.14%
conjunctival disease	14	1.24995e7	1.99366e5	5.3103	16	1.01%	87	8.58151e3	2.6628	14	38	0.57%
Mycobacterium avium complex infectious	15	1.86096e7	2.77034e5	25.6159	6	0.38%	144	4.10269e2	7.2277	3	3	0.12%
bullous keratopathy	69	1.74114e4	5.63474e3	6.1910	7	0.44%	71	1.35438e3	6.1952	6	7	0.24%
keratopathy	73	2.03712e4	6.23134e3	6.0316	7	0.44%	79	4.29430e3	5.4208	6	8	0.24%
chronic cystitis	87	3.23408e4	8.30081e3	4.3030	9	0.57%	137	3.63986e2	3.3729	7	15	0.29%
cystitis	91	3.90705e4	9.58730e3	4.1906	9	0.57%	159	4.91468e2	3.1621	7	16	0.29%
fatty liver	92	3.95790e4	9.60652e3	2.0551	28	1.76%	153	4.34378e2	1.8069	23	92	0.94%
interstitial cystitis	122	7.27274e4	1.33115e2	4.2617	8	0.50%	111	1.78515e2	4.3366	6	10	0.24%
chronic interstitial cystitis	122	7.27274e4	1.33115e2	4.2617	8	0.50%	111	1.78515e2	4.3366	6	10	0.24%
staphylococcal toxic shock syndrome	139	1.16884e3	1.87771e2	8.9366	4	0.25%	144	4.10269e2	7.2277	3	3	0.12%
membranous glomerulonephritis	182	2.64620e3	3.24669e2	3.4605	8	0.50%	88	8.73331e3	3.8548	8	15	0.33%

The test set of 1,592 genomic regions picked 2,455 genes which are differentially expressed between ALT vs PBS (14%) of all 17,744 genes., Disease Ontology has 2,233 terms covering 7,910 (45%) of all 17,744 genes. 2,233 ontology terms were tested (100%) using an annotation count range of [1, Inf].

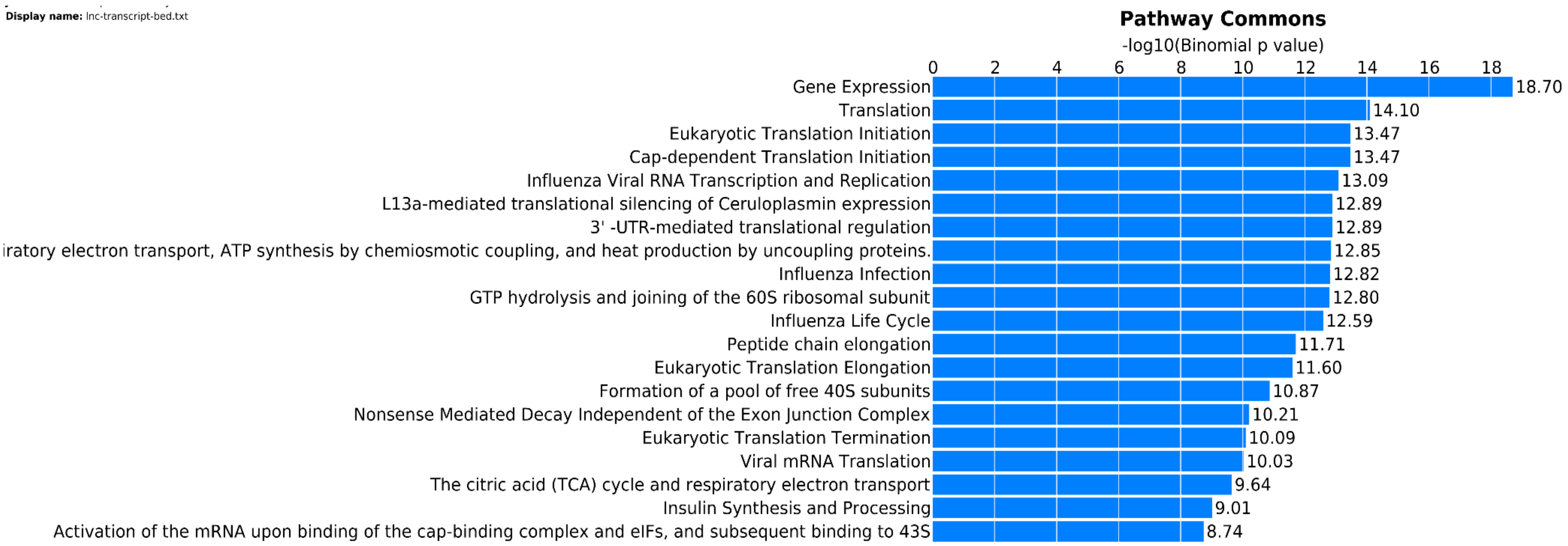

Figure S10: Disease ontology highly enriched for the deregulated mRNAs from the mRNA-lncRNAs pairs.
Genomic Regions Enrichment of Annotations Tool (GREAT) enrichment analysis of differentially regulated 1145 matched mRNA-lncRNA pairs based on pathway common GO terms. The GREAT algorithm associates each human gene (n 17,744) with a regulatory domain in the human genome (hg19 assembly) and calculates the total fraction of the genome annotated with GO terms. Listed in the figure are the most significantly enriched biological process GO terms (out of 1,607). Expected and observed counts for each GO term are listed with binomial test P value. Figure presents the top pathway common with the highest p-value. (Details in Table S9).

Table S9. Common pathways highly enriched for the deregulated mRNAs from the mRNA-lncRNAs pairs (Analysed by GREAT and presented in Figure S10).

# Term Name	Binom Rank	Binom Raw P Value	Binom FDR QVal	Binom Fold Enrichment	Binom Observed Region Hits	Binom Region Set Coverage	Hyper Rank	Hyper FDR QVal	Hyper Fold Enrichment	Hyper Observed Gene Hits	Hyper Total Genes	Hyper Gene Set Coverage
Gene Expression	1	1.99636e19	3.20815e16	2.8663	97	6.09%	50	1.59671e3	1.5286	81	383	3.30%
Translation	3	7.98788e15	4.27884e12	4.3760	42	2.64%	49	9.58771e4	2.0309	34	121	1.38%
Eukaryotic Translation Initiation	4	3.37100e14	1.35430e11	4.3794	40	2.51%	51	1.59542e3	2.0288	32	114	1.30%
Capdependent Translation Initiation	4	3.37100e14	1.35430e11	4.3794	40	2.51%	51	1.59542e3	2.0288	32	114	1.30%
Influenza Viral RNA Transcription and Replication	6	8.18308e14	2.19170e11	4.7051	36	2.26%	88	2.23416e2	1.8424	26	102	1.06%
L13amediated translational silencing of Ceruloplasmin expression	7	1.28894e13	2.95904e11	4.3976	38	2.39%	54	2.16282e3	2.0456	30	106	1.22%
3' UTRmediated translational regulation	7	1.28894e13	2.95904e11	4.3976	38	2.39%	54	2.16282e3	2.0456	30	106	1.22%
Respiratory electron transport, ATP synthesis by chemiosmotic coupling, and heat production by uncoupling proteins.	9	1.42623e13	2.54660e11	4.6148	36	2.26%	6	2.92463e4	2.5617	28	79	1.14%
Influenza Infection	10	1.52688e13	2.45369e11	3.8399	44	2.76%	90	2.43019e2	1.6832	34	146	1.38%
GTP hydrolysis and joining of the 60S ribosomal subunit	11	1.59739e13	2.33364e11	4.3660	38	2.39%	57	2.47534e3	2.0265	30	107	1.22%
Influenza Life Cycle	12	2.57216e13	3.44455e11	3.8531	43	2.70%	89	2.31032e2	1.7037	33	140	1.34%
Peptide chain elongation	13	1.95349e12	2.41481e10	4.8534	31	1.95%	65	9.46431e3	2.0408	24	85	0.98%
Eukaryotic Translation Elongation	14	2.53446e12	2.90920e10	4.8030	31	1.95%	78	1.37155e2	1.9712	24	88	0.98%
Formation of a pool of free 40S subunits	15	1.36366e11	1.46094e9	4.2328	33	2.07%	71	1.03289e2	1.9575	26	96	1.06%
Nonsense Mediated Decay Independent of the Exon Junction Complex	17	6.23441e11	5.89335e9	4.3425	30	1.88%	97	3.63728e2	1.8471	23	90	0.94%
Eukaryotic Translation Termination	19	8.04847e11	6.80731e9	4.4333	29	1.82%	98	3.73846e2	1.8707	22	85	0.90%
Viral mRNA Translation	20	9.42614e11	7.57391e9	4.4027	29	1.82%	98	3.73846e2	1.8707	22	85	0.90%
The citric acid (TCA) cycle and respiratory electron transport	23	2.27383e10	1.58871e8	3.2614	40	2.51%	47	3.45032e4	2.1819	32	106	1.30%
Insulin Synthesis and Processing	26	9.82709e10	6.07390e8	2.9951	42	2.64%	91	2.40525e2	1.7132	32	135	1.30%
Activation of the mRNA upon binding of the cap binding complex and eIFs, and subsequent binding to 43S	30	1.83600e9	9.83486e8	4.3983	25	1.57%	60	3.92576e3	2.4092	19	57	0.77%

The test set of 1,592 genomic regions picked 2,455 genes which are differentially expressed between ALT vs PBS (14%) of all 17,744 genes., Pathway Commons has 1,607 terms covering 6,064 (34%) of all 17,744 genes. 1,607 ontology terms were tested (100%) using an annotation count range of [1, Inf].

Table S10: Characteristics of the donors and properties of islets/tissues used for transplantation as well as for other experimental purposes

Donor ID/ Cat No.	Sources/ Supplier	Donor Number	Tissues/ Cells Utilized	Use/purpose	Purity of Islet	Islet Post-culture Viability	Donor Age (Year)	Donor Gender	Disease Condition	Donor BMI	Donor Height (CM)	Donor BW (KG)
ABDE148	IIDP	Donor 1	Islets	Transplantation/RNA Seq/RT-PCR	85%	95%	47	Female	Normal	22.5	152.4	52.2
ABFG183	IIDP	Donor 2	Islets	Transplantation/RNA Seq/RT-PCR	85%	97%	51	Female	Normal	25.4	160	65
ABFV308	IIDP	Donor 3	Islets	Transplantation/RNA Seq/RT-PCR	85%	97%	45	Female	Normal	27.4	167.6	77.1
ABGY290	IIDP	Donor 4	Islets	RT-PCR	90%	94%	49	Female	Normal	36.9	172.7	110
ACCZ181	IIDP	Donor 5	Islets	RT-PCR	95%	98%	19	Male	Normal	34.1	188	120
ACDL087B	IIDP	Donor 6	Islets	RT-PCR	95%	93%	42	Male	Normal	36.8	170	106.4
ABDG032	IIDP	Donor 7	Islets	RT-PCR	95%	80%	61	Male	Type 2 Diabetes	42.1	170.18	122
ABFD452	IIDP	Donor 8	Islets	RT-PCR	90%	98%	41	Male	Type 2 Diabetes	30.7	170.18	88.9
ACDB089	IIDP	Donor 9	Islets	RT-PCR	90%	97%	52	Female	Type 2 Diabetes	25.5	170	73.9
ABKO130	IIDP	Donor 10	Islets	RT-PCR	75%	95%	52	Male	Type 2 Diabetes	32.2	157.48	79.83
ACDW320A	IIDP	Donor 11	Islets	Culture/RT-PCR	90%	92%	47	Male	Normal	35.5	167.64	99.79
ACEK498	IIDP	Donor 12	Islets	Culture/RT-PCR	90%	91%	25	Male	Normal	24.3	188	86
ACEQ383A	IIDP	Donor 13	Islets	Culture/RT-PCR	90%	96%	41	Male	Normal	29.3	180.34	95.25
540019	Agilent tech	Donor 14,15,16,17,18	Adult lung	RT-PCR	N/A	N/A	19, 56, 56, 59	Male	Normal/unknown	Unknown	Unknown	Unknown
540029	Agilent tech	Donor 19	Skeletal Muscle	RT-PCR	N/A	N/A	85	Female	Normal/unknown	Unknown	Unknown	Unknown
540141	Agilent tech	Donor 20	Adult Thymus	RT-PCR	N/A	N/A	18	Male	Normal/unknown	Unknown	Unknown	Unknown
540011	Agilent tech	Donor 21	Adult Heart	RT-PCR	N/A	N/A	63	Female	Normal/unknown	Unknown	Unknown	Unknown
540013	Agilent tech	Donor 22	Adult Kidney	RT-PCR	N/A	N/A	76	Female	Normal/unknown	Unknown	Unknown	Unknown
540017	Agilent tech	Donor 23,24,25	Adult Liver	RT-PCR	N/A	N/A	30, 44, 55	Male, Female	Normal/unknown	Unknown	Unknown	Unknown
540005	Agilent tech	Donor 26	Adult Brain	RT-PCR	N/A	N/A	66	Female	Normal/unknown	Unknown	Unknown	Unknown
540035	Agilent tech	Donor 27,28	Adult Spleen	RT-PCR	N/A	N/A	45, 54	Male	Normal/unknown	Unknown	Unknown	Unknown

Table S11: List of the primers and sequences used in this study.

Sequence Name	Primer Sequence (5'-3')	Sequence Name	Primer Sequence (5'-3')	Sequence Name	Primer Sequence (5'-3')	Sequence Name	Primer Sequence (5'-3')
GTF3C5-1:1F	CCT ACT GGA CCA ACT TTG C	ARL4A-5:1R	CTC AGC TTA CCC AAC TTG TA	INSF	TAC CAG CAT CTG CTC CCT CT	RASA1-13:3R	AAA TGC TCT TCA TAT CTT CCC A
GTF3C5-1:1R	CTG CTG CCC ATC TTC TTG	CEL001F	CAA CAA GGG CAA CAA GAA AG	INSR	TGC TGG TTC AAG GGC TTT AT	UBE4A-1:4F	GCG ATC TGC TGT GAC TTA C
PIK3C2A-1:1F	AAA TGG CTC GGA CTG TG	CEL001R	GAC TCG GTG TAG ACA TCA AAG	GCKF	TGG ACC AAG GGC TTC AAG GCC	UBE4A-1:4R	CAG TCT GAG CAC TAT TGA CTA TT
PIK3C2A-1:1R	TGG CTC ACA TAT TTC TCA TGT A	RPS13001F	GAG AGG AAC AGA AAG GAT AAG G	GCKR	CAT GTA GCA GGC ATT GCA GCC	F2RL2-5:1F	GGA GCA GTG TGA AGA AGA G
RPS27-2:1F	AGA ATG GTC CAG CGT TTG	RPS13001R	GCA GAG GCT GTA GAT GAT TC	PCSK1F	TCG CGC CTC CTA GCT CTT CGC A	F2RL2-5:1R	CCT TGG CTT TAT CTC CCT TAG
RPS27-2:1R	GTG CTT TCC CAT CCA TCT T	RPS27002F	GAG GAG AAG AGG AAA CAC AAG	PCSK1R	GCA GAC TCC AGG CTC TTC GCT C	COX7B-1:2F	TAG GAG GGA CTT GAG AGA TTG
CPA5-1:1F	CTT CTA GCT TAA CCT CAA GTC C	RPS27002R	AGG ATA CGA GAC AGC TAA CA	PCSK2F	TCG ACC AGG TGG TGC GGG AT	COX7B-1:2R	GCT ACA CAA GAT GGA AAG GG
CPA5-1:1R	CCC AAA GGC CTT CTA TAA CC	PFKFB3F	CTT GCC TAC TTC CTG GAT AAG	PCSK2R	AAA GGC GGA TGT GCA GCG CT	CTRB1-1:4F	CTC CTA TCC TCC CAG TAC AA
SAG-3:1F	GAT GTC CCT CAT TCT GTG TTC	PFKFB3R	ACT CCA CGT TCA GGT AGA T	IL1R1F	CCT CCC AGG GGC TCC ACC TG	CTRB1-1:4R	GAT GAG TGT CAT TCC CTG AC
SAG-3:1R	CAG CTT GAA CAA GGC AGA T	SRPK1-1:1F	CTG ACC TGT GAG GGA GAC AAG A	IL1R1R	AGC TGG AGG ACA GGG CAG GG	CTRB1-1:1F	TGA AGT TTG GCC TCA AGT G
SERINC1-4:1F	GCT GAG ATC CCT AGA GCT ATT	SRPK1-1:1R	ATT CTG GGC TAG GTG TGG GA	IFNGR1F	CCG TCG GTA GCA GCA TGG CT	CTRB1-1:1R	TCC TCG CTG ATG TGG TAG
SERINC1-4:1R	GCC TAT GAT CTC TCC GAC ATA	RPL8-1:1F	CAT CGT CAA GGA CAT CAT CC	IFNGR1R	AGA TCC GCG GTG CCC ATC TCA	CTRB1-1:5F	AGG ATG GCT AAC GCT AAG G
SCYL1-1:22F	GAA GAG GCA ATG TCC ATC TC	RPL8-1:1R	CAA GAT GGG TTT GTC AAT TCG	TNFRSF1AF	GTC CTG CAG GGG CAA GCA GG	CTRB1-1:5R	TGG TGA CAA CAG GAG GTG
SCYL1-1:22R	CAC TTG ATC CCA ACT CAT CTC	NEMF-2:1F	AGG AGT TCT GGG CTG TAG T	TNFRSF1AR	CAG GCT CTT GAG CCC ACG GC	POLG2-1:1F	CAC TGA ACC CAC TGC TAT TC
CPNE4-2:1F	TCG TAG ACC TTG CCT GAC	NEMF-2:1R	TTT GAC CTG CTC CGT TTC C	CASP3F	TGG TTC ATC CAG TCG CTT TG	POLG2-1:1R	CAA GAG TTC TCC CAA ACT TAC A
CPNE4-2:1R	CAG GGA AAT GTT TAG AGT CCT G	NUS1-5:1F	CAA GGA GGT TAA GCC CAA GAT	CASP3R	CAT TCT GTT GCC ACC TTT CG	NT5M-3:1F	CGC CAG TAC CTG TCT TAT TG
SMC5-6:1F	GAG GAA TCC TTG GCA GAT AAA	NUS1-5:1R	CCT GGG CCT TGG TTT GAT	BAXF	CCC TTT TGC TTC AGG GTT TC	NT5M-3:1R	GCT CTG TGT AGA TGT CTT TGT
SMC5-6:1R	GCA AGA GAT GCA CCA GTA A	GAPDHF	TGC ACC ACC AAC TGC TTA	BAXR	TGT TAC TGT CCA GTT CGT CC	SERPINA5-1:2F	TCC ATC TCG AGG GAC TAT AAC
DYNC1LI1-4:1F	GGC CGC AAA GAA GAT GAA	GAPDHR	GGA TGC AGG GAT GAT GTT C	BCL2F	TGT GGA GAG CGT CAA CCG GGA G	SERPINA5-1:2R	GTT CGG ATA CAA AGG ATG AAT TG
DYNC1LI1-4:1R	CTG AGC AGT TGT TAG CAA GA	ACTBF	AGA GCC TCG CCT TTG CCG ATC C	BCL2R	ATC AAA CAG AGG CCG CAT GCT G	RPL24-3:1F	GCC ATG AAG GCT GCT AAG
ATP5I-2:1F	CTC TCC GCT CAT CAA GAT TAC	ACTBR	CAC ATG CCG GAG CCG TTG TCG	NFKB1F	CCT GGA TGA CTC TTG GGA AA	RPL24-3:1R	TTT CAC AGG CTT CAC AAT CT
ATP5I-2:1R	GCA GGG TCA CTC ACT TTA AT	HSPA4F	AGC AGC GCT CTC GGT TGC AG	NFKB1R	TCA GCC AGC TGT TTC ATG TC	SAT2-2:1F	AAG TCC GTA GGC CCT TTA
PPY-1:1F	TCT TCC CTC CCA GGT ATG	HSPA4R	AGA CAG GAC ACG GAC CCC CG	STAT1F	GCC AAA GGA AGC ACC AGA GCC AAT	SAT2-2:1R	GCA GTC TTC ACC CGA ATC
PPY-1:1R	GAG ACA GAA GGT GGC ATT AT	HSPA5F	TGC TGC TGC CCA ACT GGC TG	STAT1R	AGGAGACATGGGGAGCAGGTTGT	ARL4A-5:1F	CAT GTG TGT ACC TCC TGA AG
PLGLB2-5:1F	CCC ATA CTT CTT ACA CTA TTC CTC	HSPA5R	GAA CAC GCC GAC GCA GGA GT	DDIT3F	GGA GCA TCA GTC CCC CAC TT	BCAR1-2:1R	CAC AGG TTT GGT AGA GTG AG
PLGLB2-5:1R	CAA TGA ATG AAG CGA ACA GAT T	EIF2AF	ACG CCG CTC TTG ACA GTC CG	DDIT3R	TGT GGG ATT GAG GGT CAC ATC	RASA1-13:3F	CCA TTT CAT CTG TCC TCA TTC T
BCAR1-2:1F	AGG TTT GGG TGG TGA ATG	EIF2AR	TTG CCC CAG GCA AAC AAG GTC C				
